# Transcription factor HNF4α2 promotes osteogenesis and prevents bone abnormalities in mice with renal osteodystrophy

**DOI:** 10.1172/JCI159928

**Published:** 2023-06-01

**Authors:** Marta Martinez-Calle, Guillaume Courbon, Bridget Hunt-Tobey, Connor Francis, Jadeah Spindler, Xueyan Wang, Luciene M. dos Reis, Carolina S.W. Martins, Isidro B. Salusky, Hartmut Malluche, Thomas L. Nickolas, Rosa M.A. Moyses, Aline Martin, Valentin David

**Affiliations:** 1 Division of Nephrology and Hypertension, Department of Medicine, and Center for Translational Metabolism and Health, Institute for Public Health and Medicine, Northwestern University Feinberg School of Medicine, Chicago, Illinois, USA.; 2 LIM 16, Nephrology Department, Hospital das Clínicas da Faculdade de Medicina da USP (HCFMUSP), Universidade de São Paulo, São Paulo, Brazil.; 3 Department of Pediatrics, David Geffen School of Medicine at UCLA, Los Angeles, California, USA.; 4 Division of Nephrology, Bone and Mineral Metabolism, Department of Internal Medicine, University of Kentucky, Lexington, Kentucky, USA.; 5 Department of Medicine, Columbia Irving University Medical Center, New York, New York, USA.

**Keywords:** Bone Biology, Metabolism, Bone disease, Chronic kidney disease

## Abstract

Renal osteodystrophy (ROD) is a disorder of bone metabolism that affects virtually all patients with chronic kidney disease (CKD) and is associated with adverse clinical outcomes including fractures, cardiovascular events, and death. In this study, we showed that hepatocyte nuclear factor 4α (HNF4α), a transcription factor mostly expressed in the liver, is also expressed in bone, and that osseous *HNF4α* expression was dramatically reduced in patients and mice with ROD. Osteoblast-specific deletion of *Hnf4α* resulted in impaired osteogenesis in cells and mice. Using multi-omics analyses of bones and cells lacking or overexpressing *Hnf4α1* and *Hnf4α2*, we showed that HNF4α2 is the main osseous *Hnf4α* isoform that regulates osteogenesis, cell metabolism, and cell death. As a result, osteoblast-specific overexpression of *Hnf4α2* prevented bone loss in mice with CKD. Our results showed that HNF4α2 is a transcriptional regulator of osteogenesis, implicated in the development of ROD.

## Graphical abstract

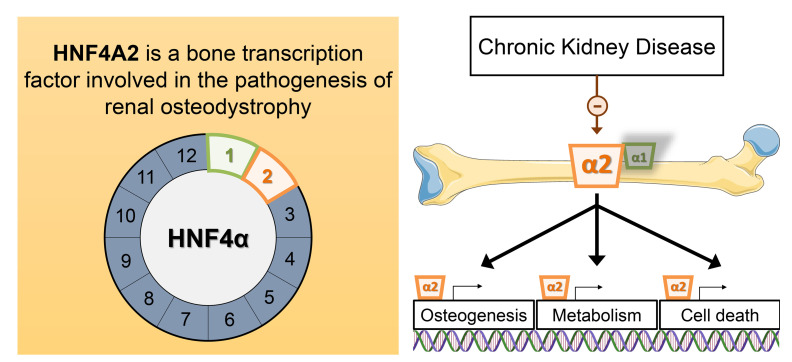

## Introduction

Chronic kidney disease (CKD) is a costly public health burden that increases the risk of mortality (1). Disordered bone and mineral metabolism is a nearly universal complication of CKD, collectively termed CKD–mineral and bone disorder (CKD-MBD), that begins early and worsens progressively as kidney function declines (2–4).

Renal osteodystrophy (ROD) is the bone disease associated with CKD. ROD is a disorder of bone cell function and metabolism that leads to abnormal structure and compromised bone strength. Loss of bone quantity and quality due to high and low bone turnover and further onset of bone lesions are strongly associated with progressive impairment of kidney function. The exact pathogenesis of ROD is poorly understood, but it is often described as a particular subset of metabolic bone disease. Although disturbances in circulating factors, such as calcitriol, parathyroid hormone, and fibroblast growth factor 23, and the resulting impact on phosphate and calcium levels, have major skeletal effects, in recent years it became clear that intrinsic osseous mechanisms might contribute to the onset and progression of ROD (5–7). Indeed, skeletal abnormalities persist despite therapy with different active vitamin D sterols and phosphate binders (8–10), and bone deformities, fractures, and growth retardation remain the long-term consequences of CKD for the growing skeleton (11–13). To date, the molecular mechanisms of bone loss in ROD remain to be determined.

Hepatocyte nuclear factor 4α (HNF4α) is a highly conserved transcription factor, a member of the nuclear receptor (NR) family, which regulates gene transcription by binding DNA as a dimer. In contrast to other types of NR, HNF4α is constitutively localized in the nucleus and does not require binding of a ligand to homodimerize and interact with the response elements of its target genes (14). HNF4α can function as an activator or repressor of genes involved in cell metabolic activity, transport, glucose and lipid homeostasis, and detoxification of xenobiotics (15–20). HNF4α was initially discovered as a regulator of liver-specific gene expression. However, HNF4α expression has also been described in multiple other organs, including pancreas, kidney, stomach, small intestine, and colon (19, 21–23). Mutations of *HNF4α* and HNF4α response elements cause maturity-onset diabetes of the young 1 (MODY1), a rare disease; certain types of hemophilia; and hepatitis B viral infections. In addition, HDL-cholesterol (24), metabolic dyslipidemia (25), and type 2 diabetes mellitus (26, 27) have been associated with the HNF4α locus by genome-wide associations studies. Importantly, HNF4α is also associated with coronary artery calcification in the Chronic Renal Insufficiency Cohort (28) and with osteoporosis in the Framingham Osteoporosis Study (29), which was mostly attributed to HNF4α function in liver and kidney. However, the direct role of HNF4α in bone has never been investigated despite the clear associations between HNF4α and disturbances in bone and mineral metabolism.

In the present study, we report the expression of 2 main isoforms of HNF4α in bone, HNF4α1 and HNF4α2, and we investigated the role of osseous HNF4α in the pathology of ROD in human and experimental models. First, we show that HNF4α expression is nearly completely suppressed in bone from patients and mice with CKD. We report that HNF4α2 is a major regulator of osteogenesis using genetics and multi-omics approaches in vitro and in vivo. Finally, we show the key impact of restoring osteoblastic HNF4α2 expression on bone mass in mice with CKD. These results establish the direct role of osseous HNF4α2 in the regulation of osteogenesis, suggest that osseous HNF4α2 deficiency contributes to the pathogenesis of ROD, and propose a mechanism to explain intrinsic bone defects in patients with CKD.

## Results

### HNF4α is expressed in bone and its expression is reduced in patients and animals with CKD.

**Figure 1:**
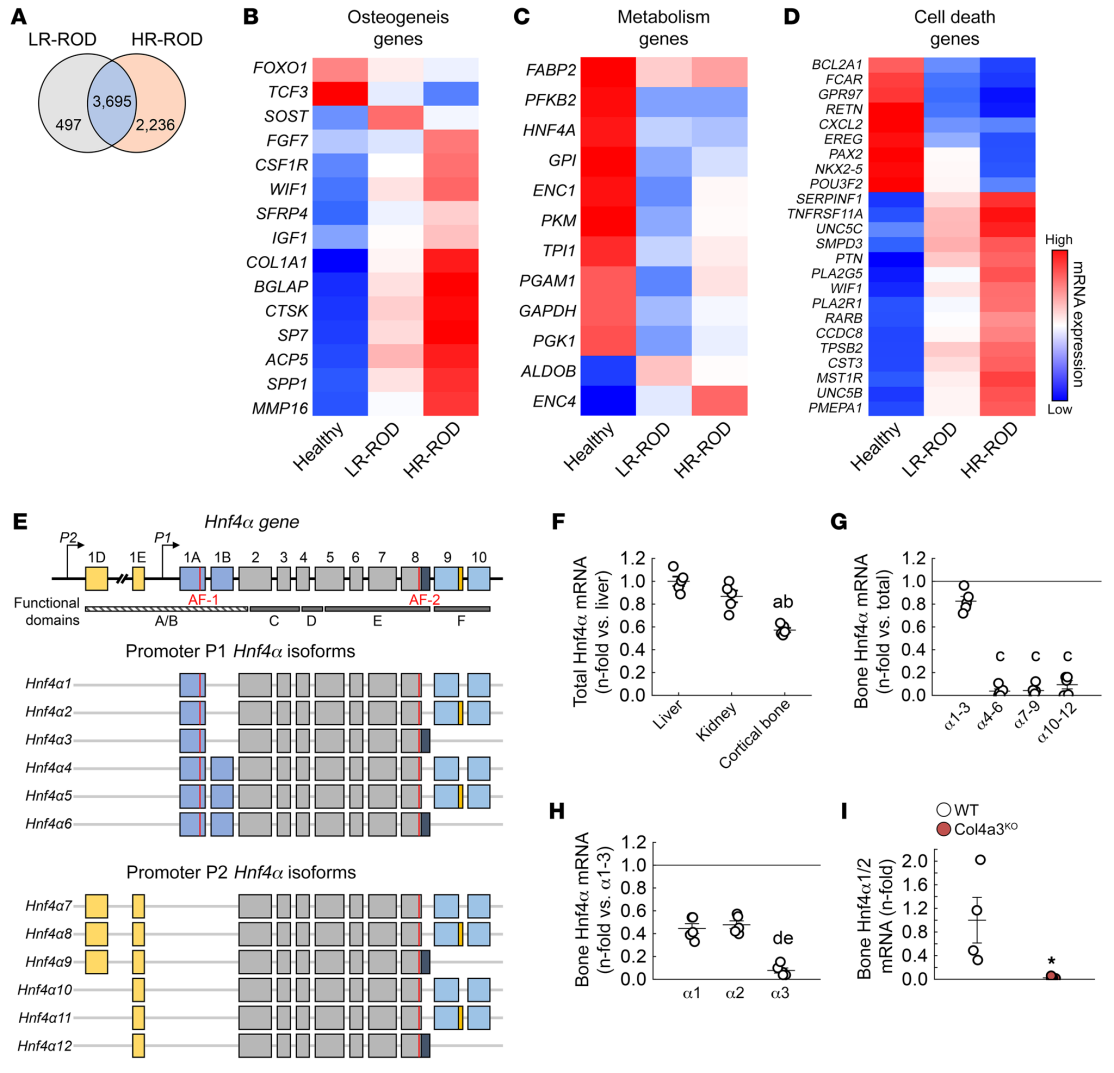
HNF4A is expressed in bone and is reduced in humans and mice with CKD. (**A**) Number of differentially regulated genes identified by RNA-Seq of bone biopsies from CKD patients with low–bone remodeling (LR) and high–bone remodeling (HR) renal osteodystrophy (ROD) versus healthy volunteers. (**B**–**D**) Heatmap-represented expression of genes identified in the topmost differentially regulated pathways in LR-ROD and HR-ROD bone biopsies versus healthy volunteers. *n* = 9 (Healthy and LR-ROD) and 11 (HR-ROD); corrected *P* < 0.05. Statistical analysis was performed with an ANOVA test followed by unpaired Student’s *t* test and corrected by the FDR. (**E**) Schematic representation of *Hnf4α* gene and different promoter P1– and P2–driven *Hnf4α* isoforms. (**F**–**H**) Comparative analysis of total *Hnf4α* mRNA in liver, kidney, and bone (**F**), *Hnf4α* isoforms 1 to 12 mRNA in bone (**G**), and *Hnf4α* isoforms 1 to 3 mRNA in bone of WT mice (**H**). (**I**) mRNA expression of *Hnf4α1/2* in bone of WT and Col4a3^KO^ mice with CKD. Values are expressed as the mean ± SEM. *N* = 5 per group. Corrected *P* < 0.05 versus ^a^liver, ^b^kidney, ^c^*Hnf4α1–3*, ^d^*Hnf4α1*, ^e^*Hnf4α2*, and *WT. Statistical analysis was performed with an unpaired Student’s *t* tests (**I**) or with an ANOVA followed by post hoc *t* tests to determine statistical differences and multiple-testing correction using the Holm-Bonferroni method (**F**–**H**).

We performed RNA-Seq on bone biopsies collected from patients (Supplemental Table 1; supplemental material available online with this article; https://doi.org/10.1172/JCI159928DS1) with or without ROD (GEO GSE194056), showing either low or high bone turnover to identify ROD-specific transcriptomic profiles (Figure 1A). We mainly identified alterations in expression of genes involved in osteogenesis, metabolism, and cell death (Figure 1, B–D). Among the metabolic genes, we identified *HNF4A*, a gene not known for its osseous expression, as a gene suppressed in all ROD patient groups compared with non-ROD patients, irrespectively of their bone remodeling status. In humans and mice, *HNF4A* encodes 12 annotated isoforms with distinct molecular functions and varying transcriptional regulatory potentials (Figure 1E). Accordingly, 12 distinct HNF4α proteins have been annotated in both humans and mice (30, 31). HNF4A isoforms are generated under the control of 2 alternative promoters, P1 and P2, which results in over 60 potential HNF4A homo- or heterodimer isoforms with different impacts on gene expression regulation (30). In adult mice, total *Hnf4α* mRNA expression was highest in liver. In comparison, the expression of total *Hnf4α* mRNA was only 40% lower in osteoblast- and osteocyte-enriched bone fraction alone (Figure 1F). Analysis of mRNA expression of the 12 annotated *Hnf4α* isoforms in mouse bones showed that isoforms 1–3 were the most represented of all *Hnf4α* isoforms, as in adult liver and kidney (32), and further analysis identified *Hnf4α1* and *Hnf4α2* as the predominant osseous isoforms (Figure 1, G and H). We next used the Col4a3^KO^ mouse model of progressive CKD, which recapitulates most of the typical features of human CKD, including ROD (4, 33, 34), to investigate changes in *Hnf4α* expression. As in patients with ROD, we found that expression of *Hnf4α1/2* was nearly completely suppressed in the bone of the Col4a3^KO^ mice (Figure 1I).

### HNF4α2 regulates osteoblastogenesis and osteoblast metabolism.

**Figure 2:**
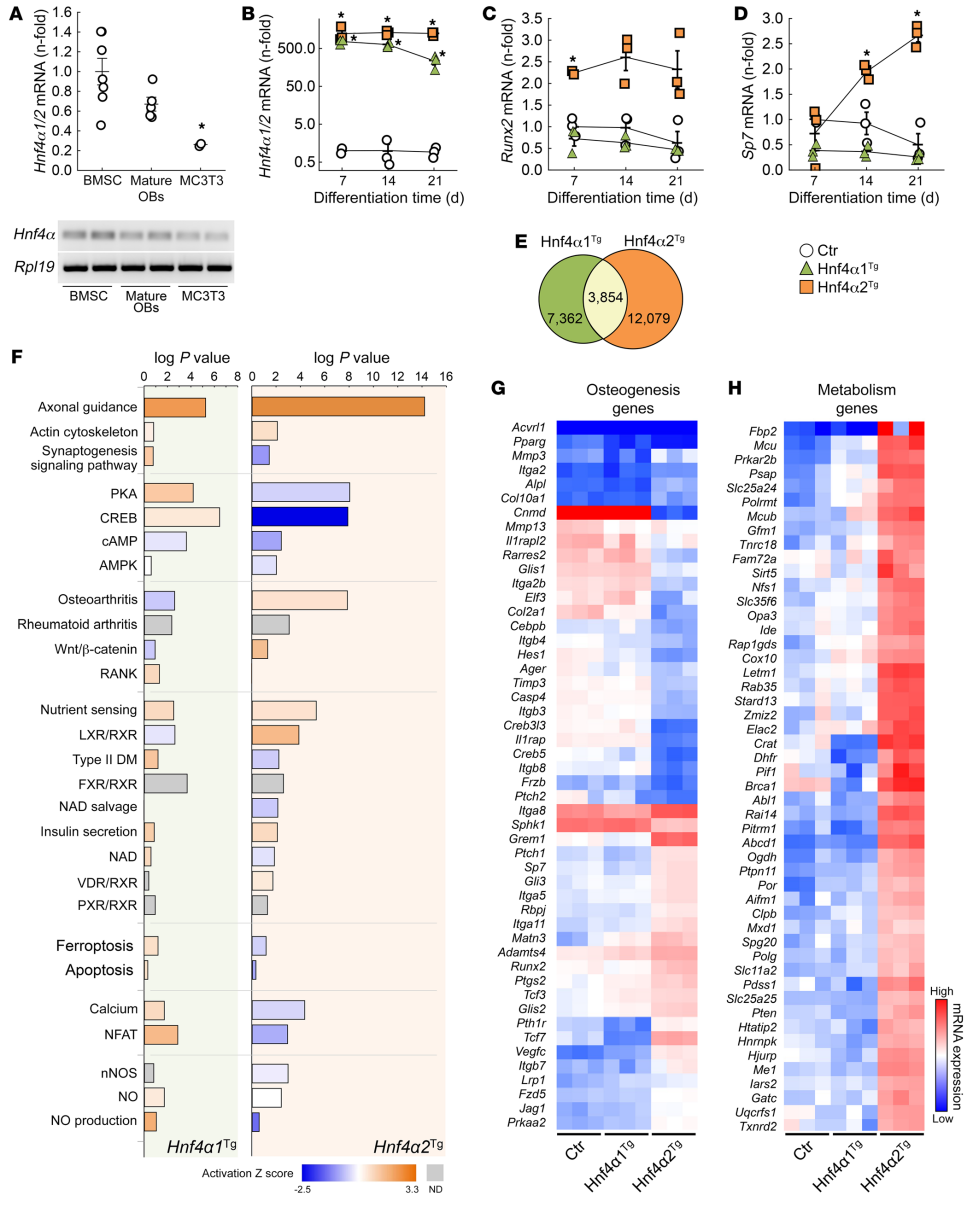
HNF4α2 is a major regulator of osteogenesis and metabolism in osteoblasts. (**A** and **B**) *Hnf4α1/2* mRNA expression in differentiated primary bone marrow stromal cells (BMSCs), mature osteoblasts (OBs), and MC3T3-E1 osteoblasts (**A**), and in MC3T3-E1 osteoblasts transfected with an empty vector (Ctr), *Hnf4α1* (Hnf4α1^Tg^), and *Hnf4α2* (Hnf4α2^Tg^) expression transgene (**B**). (**C** and **D**) mRNA expression of markers of osteoblast differentiation *Runx2* and *Sp7*. Values are expressed as the mean ± SEM. *n* ≥ 3 per group of a representative experiment performed at least 3 times; corrected *P* < 0.05 versus *BMSC or Ctr. Statistical analysis was performed with an ANOVA test followed by post hoc *t* tests to determine statistical differences and multiple-testing correction using the Holm-Bonferroni method. (**E**) Number of differentially regulated genes identified by RNA-Seq in Hnf4α1^Tg^ and Hnf4α2^Tg^ osteoblasts versus Ctr. (**F**) Canonical pathway analysis and prediction of pathway activation of differentially regulated genes identified by RNA-Seq of Ctr, Hnf4α1^Tg^, and Hnf4α2^Tg^ osteoblasts. (**G** and **H**) Heatmap-represented expression of genes modified and involved in osteogenesis and metabolism pathways in Ctr, Hnf4α1^Tg^, and Hnf4α2^Tg^ osteoblasts. Corrected *P* < 0.05; *n* = 3 per group. Statistical analysis was performed with an unpaired Student’s *t* test and corrected by the FDR.

*Hnf4α1/2* isoforms were expressed in bone marrow stromal cells (BMSCs) and primary osteoblasts, and to a lesser extent in the MC3T3-E1 osteoblast cell line, cultured for 21 days in osteogenic medium (Figure 2A). To identify the specific role of HNF4α1 and HNF4α2 in osteoblast differentiation, we overexpressed *Hnf4α1* and *Hnf4α2* in MC3T3-E1 osteoblasts (*Hnf4α1^Tg^* and *Hnf4α2^Tg^*, respectively). Compared with empty vector–transfected (Ctr) MC3T3-E1 osteoblasts, *Hnf4α* expression was about 500 times higher in both transgenic cell lines (Figure 2B). Interestingly, overexpression of *Hnf4α2*, but not *Hnf4α1*, increased expression of osteoblastic markers such as *Runx2* and *Sp7*, suggesting a major role for *Hnf4α2* in osteoblastogenesis (Figure 2, C and D). RNA-Seq (GSE190315) and subsequent pathway analyses of MC3T3-E1 Ctr, *Hnf4α1^Tg^*, and *Hnf4α2^Tg^* cells showed that overexpression of each isoform modified the expression of known HNF4α targets (Supplemental Figure 1). In addition, *Hnf4α2^Tg^* cells displayed increased cell cytoskeleton remodeling pathways, osteogenesis, and metabolic signaling and reduced cAMP/PKA signaling, cell death, calcium/NFAT, and nitric oxide pathways compared with Ctr cells. However, overexpression of *Hnf4α1* showed either a milder or an opposite effect on these pathways (Figure 2, E and F), suggesting that HNF4α1 and HNF4α2 functions are non-redundant. Importantly, *Hnf4α2^Tg^* cells showed highly modified gene expression profiles of osteogenic and metabolic markers, compared with Ctr and *Hnf4α1^Tg^* cells (Figure 2, G and H), supporting a specific role of HNF4α2 in osteoblastogenesis.

### HNF4α2 is a direct transcriptional regulator of osteoblastic genes.

**Figure 3:**
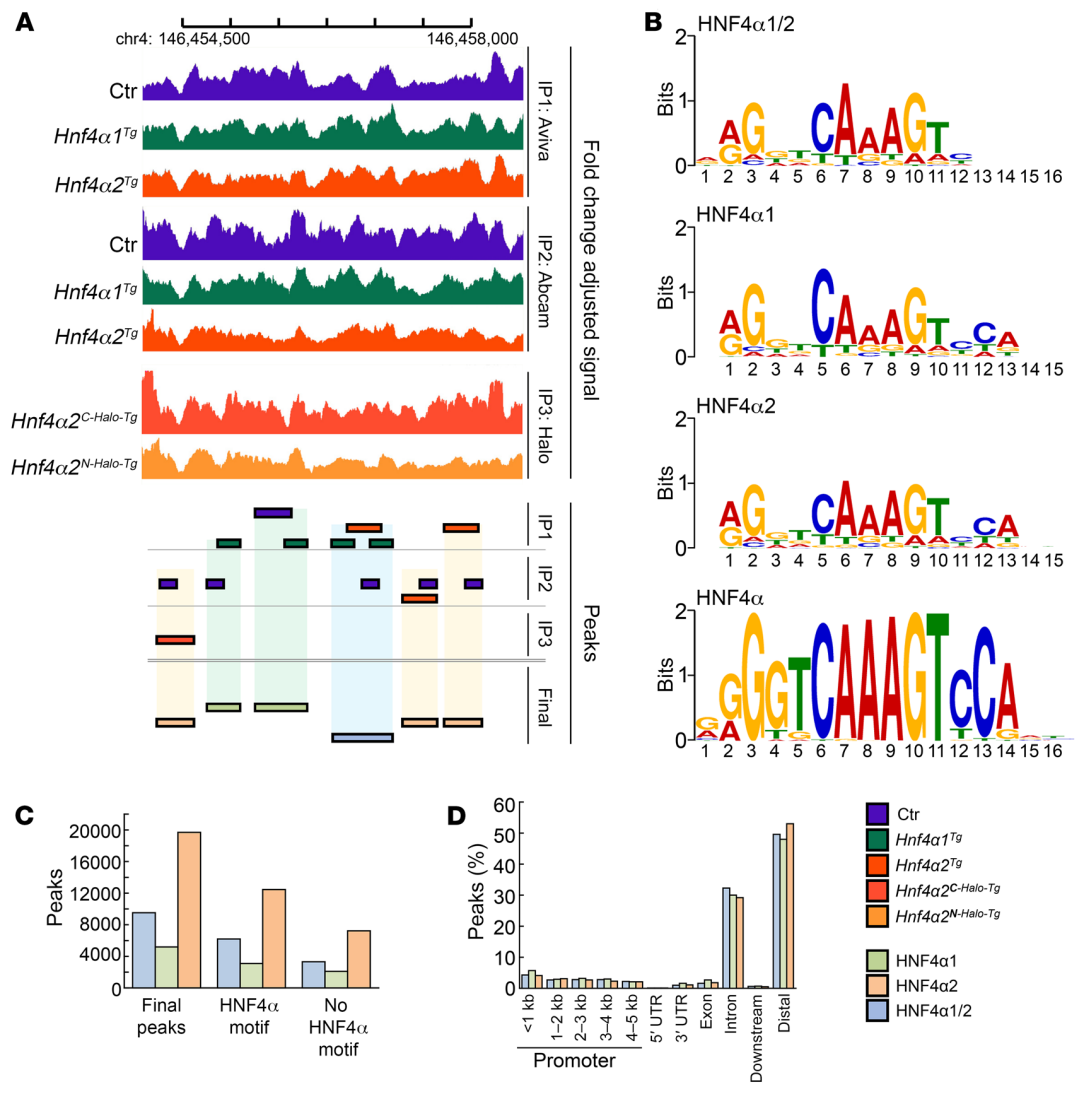
HNF4α-specific ChIP sequencing analysis of HNF4α targets in MC3T3-E1 osteoblasts. (**A**) Representative illustration of final peak calls based on overlapping naive peaks found in MC3T3-E1 osteoblasts overexpressing an empty vector (Ctr), *Hnf4α1* (Hnf4α1^Tg^), or *Hnf4α2* (Hnf4α2^Tg^). (**B**) Enriched HNF4α motif sequences found in final peaks from position frequency matrices using MEME Suite (https://meme-suite.org/meme/tools/meme-chip) compared with the curated HNF4α consensus motif. (**C** and **D**) Number (**C**) and distribution across genomic regions (**D**) of HNF4α1, HNF4α2, or common HNF4α1/2 final peaks. *n* = 3 biological replicates per experimentally used antibody.

**Figure 4:**
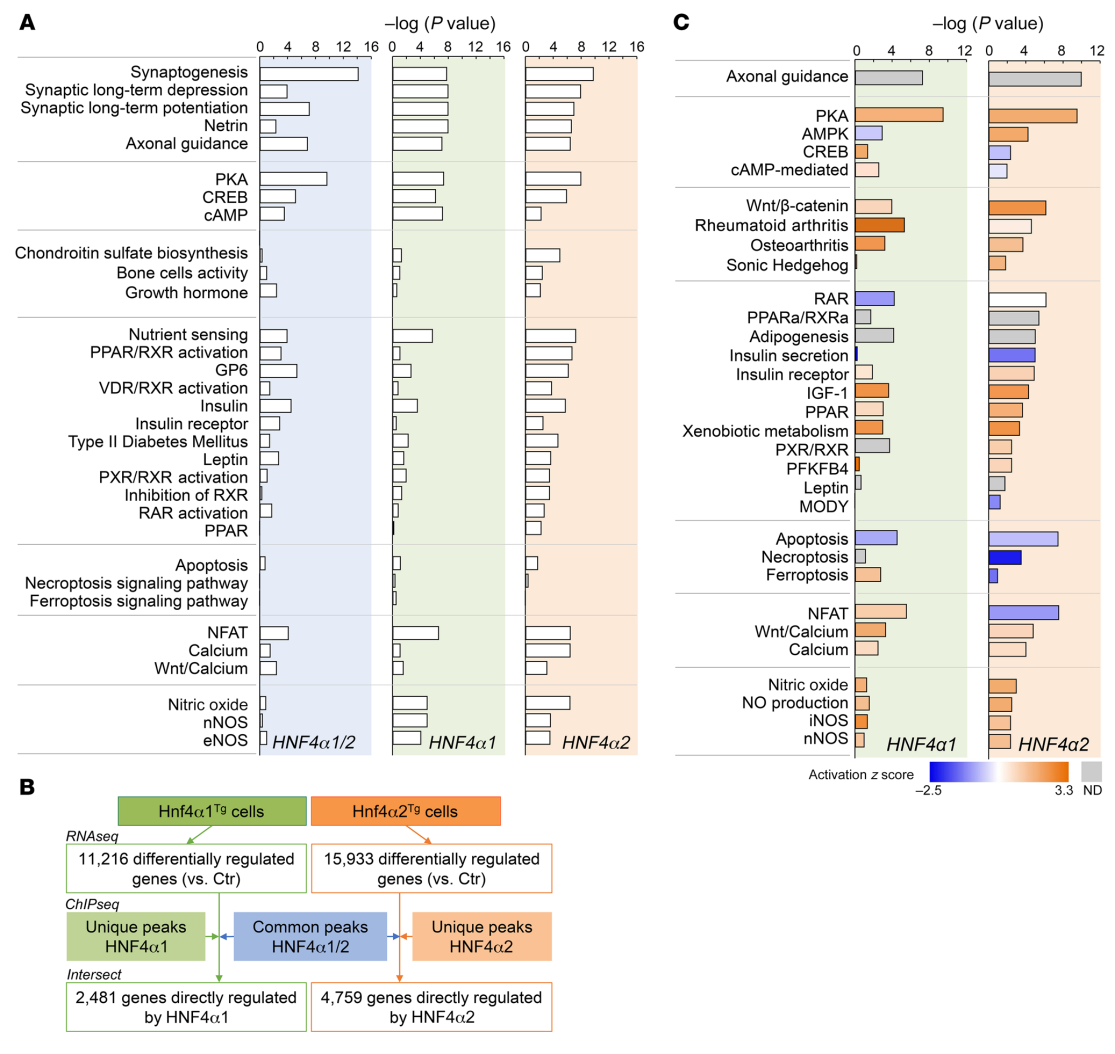
HNF4α2 is a direct transcriptional regulator of osteogenesis and metabolism in osteoblasts. (**A**) Canonical pathway analysis of HNF4α targets identified by ChIP sequencing of Ctr, Hnf4α1^Tg^, and Hnf4α2^Tg^ osteoblasts. *n* = 3 biological replicates per experimentally used antibody. (**B**) Number of genes differentially regulated in Hnf4α1^Tg^ and Hnf4α2^Tg^ osteoblasts versus Ctr and directly regulated by HNF4α, obtained from the intersection between genes identified by RNA-Seq in Figure 2 and genes identified by ChIP sequencing in Figure 3. (**C**) Canonical pathway analysis and prediction of pathway activation of direct HNF4α targets identified in **A**.

To identify genes directly regulated by HNF4α, we performed 3 different sets of chromatin immunoprecipitation sequencing (ChIP-Seq) analyses using 3 separate antibodies (GSE190314). We first performed HNF4α immunoprecipitation in Ctr, *Hnf4α1^Tg^*, and *Hnf4α2^Tg^* cell extracts using 2 different polyclonal anti-HNF4α antibodies purchased from Aviva Systems Biology and Abcam, respectively. In parallel, we generated 2 stable cell lines overexpressing *Hnf4α2* coupled with a carboxy-terminal (Hnf4α2^C-Halo-Tg^) or amino-terminal (Hnf4α2^N-Halo-Tg^) Halo tag and used an anti-Halo antibody to immunoprecipitate HNF4α. Peaks were called in each separate experiment and consolidated as follows: common HNF4α1/2 peaks resulting from the intersection of samples overexpressing either *Hnf4α1* or *Hnf4α2*; HNF4α1 peaks resulting from the intersection of 2 or more experiments overexpressing *Hnf4α1* and/or Ctr cells; HNF4α2 peaks resulting from the intersection of 2 or more experiments overexpressing *Hnf4α2* and/or Ctr cells (Figure 3A). For all chromatin immunoprecipitations, several HNF4α motifs were identified as the primary target (Figure 3B). HNF4α2 peaks were the most abundant in osteoblasts, and a majority of peaks (60%–70%) showed the expected HNF4α motif. However, a relatively large number of peaks remained without a match to the consensus motif (Figure 3C), as previously shown (35), suggesting that HNF4α binds DNA either through other motifs or by interacting with other cofactors. Consistent with prior reports, both HNF4α1 and HNF4α2 showed a preferential binding to intronic (~30%) and distal intergenic regions (~50%), with a small proportion (~10%) at gene promoters (17) (Figure 3D). Similar to results obtained in RNA-Seq analyses of *Hnf4α1^Tg^* and *Hnf4α2^Tg^* osteoblasts (Figure 2), downstream analyses of gene targets identified by ChIP-Seq showed enrichment of cell cytoskeleton remodeling, cAMP/PKA signaling, osteogenesis, metabolic, cell death, calcium/NFAT, and nitric oxide pathways (Figure 4A). Therefore, to determine whether the genes dysregulated in *Hnf4α1^Tg^* and *Hnf4α2^Tg^* osteoblasts are directly regulated by HNF4α binding to DNA, we intersected the HNF4α cistrome with the transcriptomic analyses performed in Figure 2. We found that about 2,500 genes were directly regulated by HNF4α1 and about 5,000 by HNF4α2 (Figure 4B). Downstream pathway analysis of these genes showed an enrichment in cAMP/PKA signaling, osteogenesis, metabolic, cell death, calcium/NFAT, and nitric oxide signaling pathways, supporting the important finding that HNF4α2 directly controls a large part of the osteoblast metabolic activity, differentiation, and death (Figure 4C).

### Osteoblast-specific deletion of Hnf4α reduces peak bone mass in mice.

**Figure 5:**
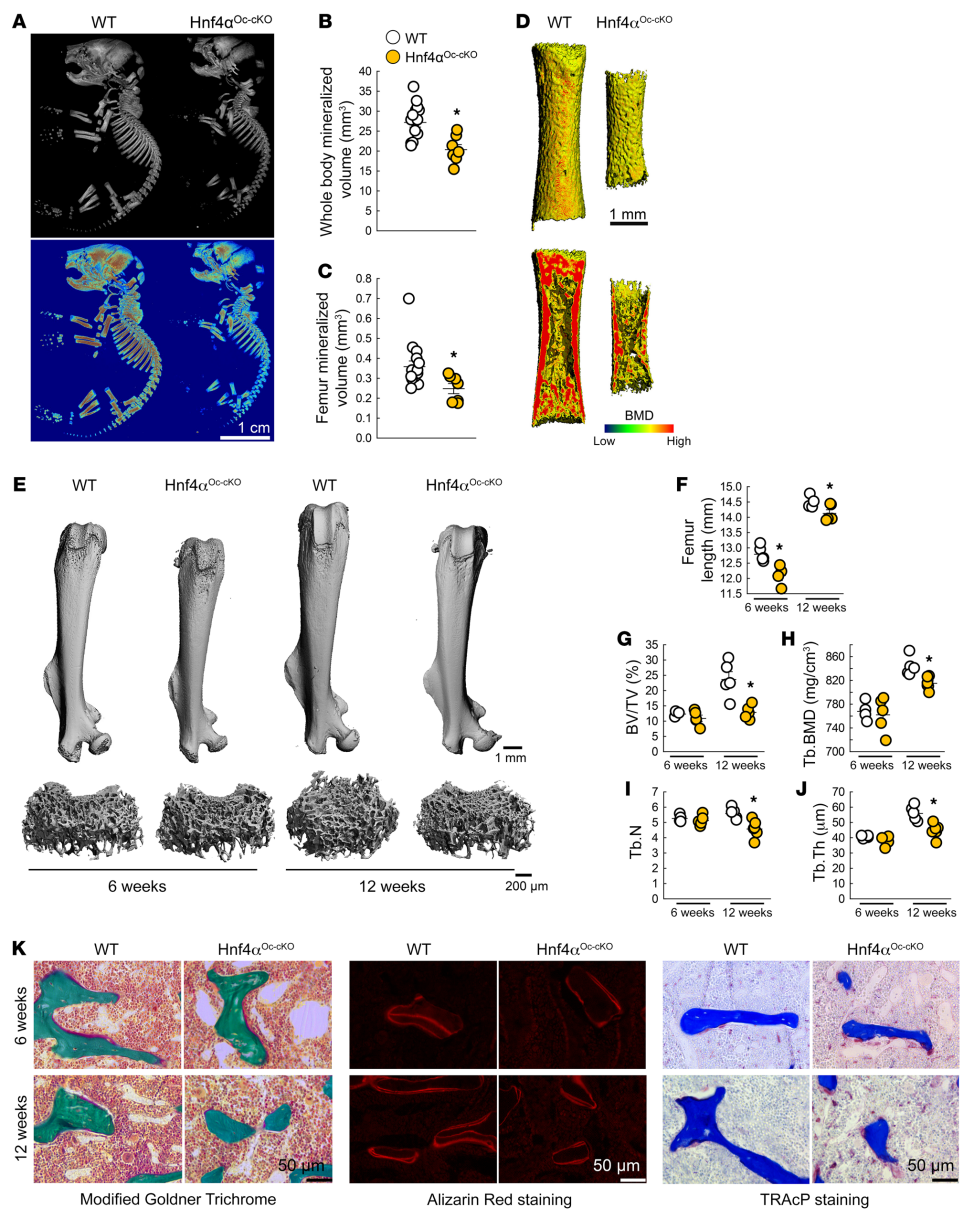
Bone-specific deletion of *Hnf4α* leads to low bone mass and impaired bone growth. (**A**–**J**) 3D microtomography analysis of whole-body skeleton (**A** and **B**) and entire femur (**C** and **D**; bottom panel of **D** shows a longitudinal section) of WT and Hnf4α^Oc-cKO^ neonates, and of entire femur and femur metaphysis of young (6 weeks) and adult (12 weeks) WT and Hnf4α^Oc-cKO^ mice (**E**–**J**). BMD, bone mineral density; BV, bone volume; TV, total volume; Tb, trabecular; N, number; Th, thickness. (**K**) Microscopy analysis of modified Goldner Trichrome staining (left), alizarin red S staining (middle), and TRAcP staining (right) of femur trabecular bone from 6- and 12-week-old WT and Hnf4α^Oc-cKO^ mice. Values are expressed as the mean ± SEM. *n* ≥ 5 per group; *P* < 0.05 versus *age-matched WT. Statistical analysis was performed with unpaired Student’s *t* tests.

Next, to determine the physiological importance of HNF4α in bone, we deleted HNF4α in osteoblasts and osteocytes (Hnf4α^Oc-cKO^). These mice showed an approximately 80% reduction in osseous *Hnf4α* expression (Supplemental Figure 2A). Hnf4α^Oc-cKO^ neonates were smaller and hypomineralized (Figure 5A) compared with their WT littermates, and showed an approximately 30% reduction in whole-body (Figure 5B) or femur (Figure 5, C and D) mineralized volume. Young and adult Hnf4α^Oc-cKO^ male mice displayed a reduction in body weight (Supplemental Figure 2B) and femur (Figure 5, E and F), tibia, and limb lengths (Supplemental Figure 2, C and D) and did not show modifications of the femur microarchitecture in cortical bone at 6 or 12 weeks of age (Supplemental Figure 2, E, D, and J). However, osteoblast-specific deletion of *Hnf4α* resulted in an approximately 50% loss of trabecular peak bone mass in 12-week-old male mice, as shown by reduced trabecular bone volume, number, and thickness and reduced trabecular bone mineral density (Figure 5, E and G–J). We observed similar changes in Hnf4α^Oc-cKO^ female mice (Supplemental Figure 3, A–E), but female mice also showed a reduction in cortical thickness and cortical area at 6 and 12 weeks of age (Supplemental Figure 3, F–J). Both male and female mice showed a reduced osteoid apposition as measured on Goldner Trichrome–stained nondecalcified bone sections and a lower bone formation rate as assessed by the reduced number of alizarin red–stained mineral seams and distance between the seams, coupled with an increase in osteoclastogenesis as shown by an increase in TRAcP-positive cells (Figure 5K and Supplemental Table 2). Notably, deletion of *Hnf4α* earlier in the osteoblastic lineage, using an Osterix-Cre–mediated deletion, exacerbated these changes in 12-week-old animals, in both male and female mice (Supplemental Figure 4).

**Figure 6:**
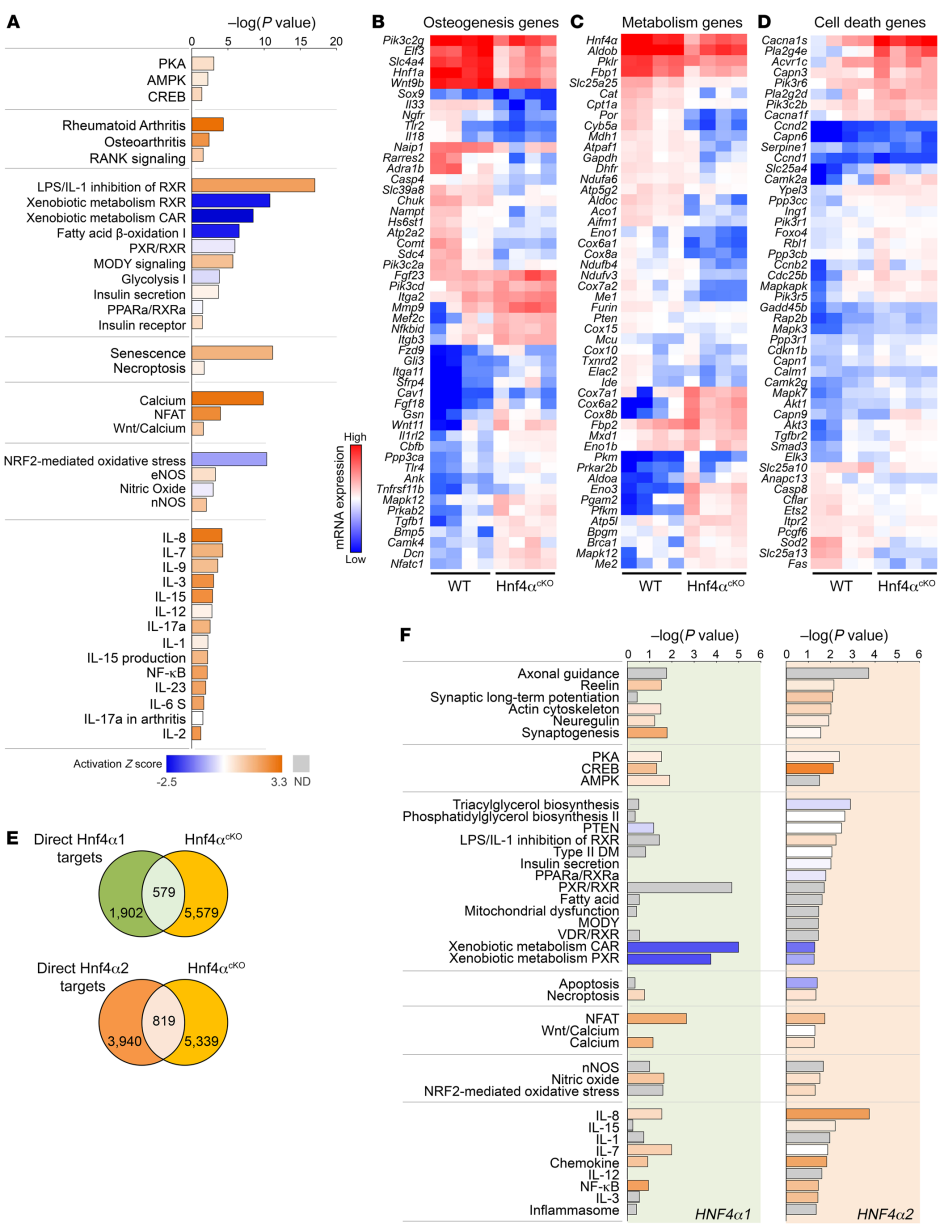
Low bone mass is associated with altered osteogenesis and impaired bone metabolism in Hnf4α^Oc-cKO^ mice. (**A**) Canonical pathway analysis of differentially regulated genes identified by RNA-Seq of bone from 6-week-old Hnf4α^Oc-cKO^ mice versus WT. (**B**–**D**) Heatmap-represented, log-normalized expression of genes identified in the topmost differentially regulated pathways in Hnf4α^Oc-cKO^ bone versus WT. (**E**) Number of genes differentially regulated in bone in Hnf4α^Oc-cKO^ versus WT identified by RNA-Seq and directly regulated by HNF4α1 or HNF4α2, obtained from the intersection with previously identified direct HNF4α targets in osteoblast ChIP sequencing in Figure 4B. (**F**) Canonical pathway analysis of direct HNF4α1 and HNF4α2 gene targets in bone identified in **E**. In **A** and **F**, prediction of pathway activation is indicated by *z* score on the heatmap. *n* = 4 per group; corrected *P* < 0.05 versus WT. Statistical analysis was performed with unpaired Student’s *t* tests and corrected by the FDR.

To determine the impact of osteoblast-specific deletion of *Hnf4α* on the expression of bone transcripts, we performed RNA-Seq on femora isolated from 6-week-old WT and Hnf4α^Oc-cKO^ male littermates (GSE190313). First, we show that reduction of *Hnf4α* in osteoblasts affected the expression of gene targets of HNF4α previously established in other tissues (Supplemental Figure 5). In addition, bones from Hnf4α^Oc-cKO^ mice showed impaired expression of genes from the major pathways identified in cultured osteoblasts (Figure 6A), leading to a defect in osteogenesis, metabolic, and cell death transcripts (Figure 6, B–D), consistent with profiles observed in patients with ROD. Interestingly, deletion of *Hnf4α* in osteoblasts increased the proinflammatory signaling in the bone, leading to activation of major cytokine signaling and prototypical NF-κB signaling (Figure 6A). Intersection of significantly altered genes in the bone of Hnf4α^Oc-cKO^ mice with transcripts directly regulated by either HNF4α1 or HNF4α2 (Figure 4B) in MC3T3-E1 osteoblast cultures identified 579 and 819 genes directly regulated by HNF4α1 and HNF4α2, respectively, in mouse bones (Figure 6E). Subsequent pathway analyses of these transcripts showed that HNF4α2 controlled cytoskeleton remodeling, metabolic, and proinflammatory signaling in bone, whereas HNF4α1 had a milder effect on these pathways, consistent with a different metabolic role (Figure 6F). In aggregate, these data demonstrate the critical role of HNF4α in bone development and structure, mediated mainly by the regulatory effects of HNF4α2 on the transcription of osteogenic, metabolic, and apoptotic gene targets.

### Hnf4α deletion in osteoblasts reduces osteoblast activity and function.

**Figure 7:**
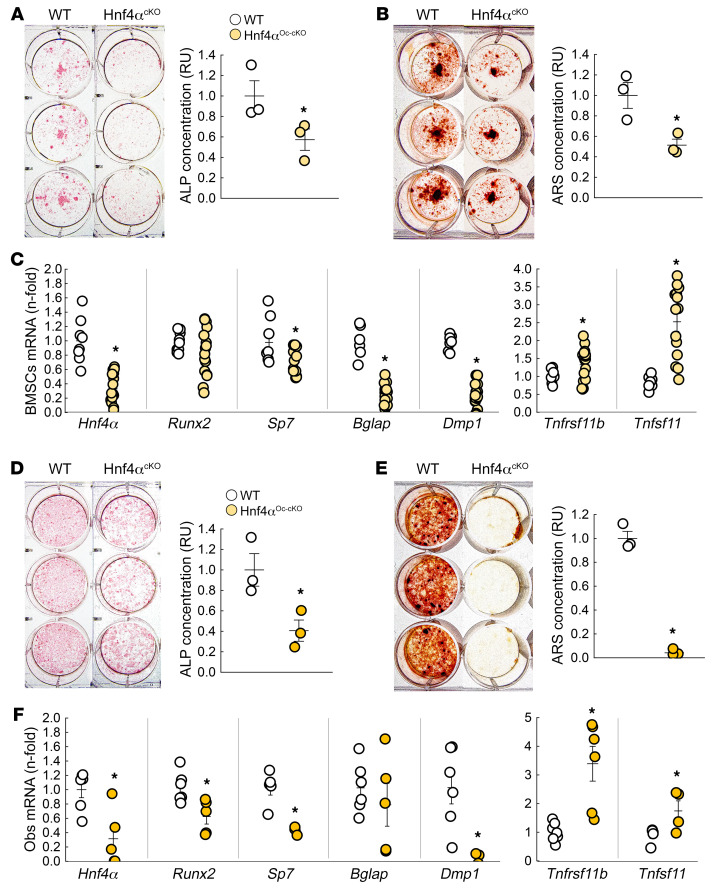
Osteoblast-specific deletion of *Hnf4α* alters osteoblast differentiation and function. (**A**–**C**) Alkaline phosphatase (ALP) (**A**) and alizarin red S (ARS) (**B**) staining and quantification and mRNA expression of *Hnf4α* and osteoblastic markers (**C**) in differentiated primary BMSC cultures isolated from 6-week-old WT and Hnf4α^Oc-cKO^ mice. (**D**–**F**) ALP (**D**) and ARS (**E**) staining and quantification and mRNA expression of *Hnf4α* and osteoblastic markers (**F**) in differentiated primary mature osteoblast cultures isolated from 6-week-old WT and Hnf4α^Oc-cKO^ mice. Values are expressed as the mean ± SEM. *n* ≥ 3 per group of a representative experiment performed at least 3 times; *P* < 0.05 versus *WT. Statistical analysis was performed with an unpaired Student’s *t* test.

To demonstrate the intrinsic role of HNF4α in osteoblast differentiation and metabolism, we isolated BMSCs and mature osteoblasts from WT and Hnf4α^Oc-cKO^ littermates and cultured them for 3 weeks in osteogenic medium. After 3 weeks, Hnf4α^Oc-cKO^ BMSC cultures showed impaired differentiation, assessed by reduced alkaline phosphatase staining (Figure 7A), and mineralization, assessed by reduced alizarin red staining (Figure 7B). As expected, Hnf4α^Oc-cKO^ BMSCs showed reduced *Hnf4α* mRNA expression (–60% vs. WT), together with reduced expression of osteogenic markers *Sp7*, *Bglap*, and *Dmp1*, supporting impaired osteoblastogenesis (Figure 7C). Interestingly, *Hnf4α* deletion led to a mild increase in the *Tnfrsf11b* gene, encoding osteoprotegerin (OPG), the decoy receptor for receptor activator of NF-κB ligand (RANKL), and a pronounced increase in *Tnfsf11*, encoding RANKL. This suggests that *Hnf4α* deletion also regulates osteoblast-induced osteoclastogenesis, consistent with observations made in vivo (Figure 5K). Notably, Hnf4α^Oc-cKO^ mature osteoblast cultures showed similar overall trends with more pronounced effects on osteoblastogenesis markers (Figure 7, D–F). The metabolomic profile of osteoblasts showed that *Hnf4α* deletion severely altered production of metabolites at the crossroads of gluconeogenesis, glycolysis, and energy metabolism (Supplemental Figure 6), and led to a reduction in NADP^+^ and NAD^+^ In aggregate, these data demonstrate that HNF4α directly controls osteoblast metabolism, differentiation, and function.

### Bone Hnf4α expression is reduced in response to acute and chronic inflammation.

**Figure 8:**
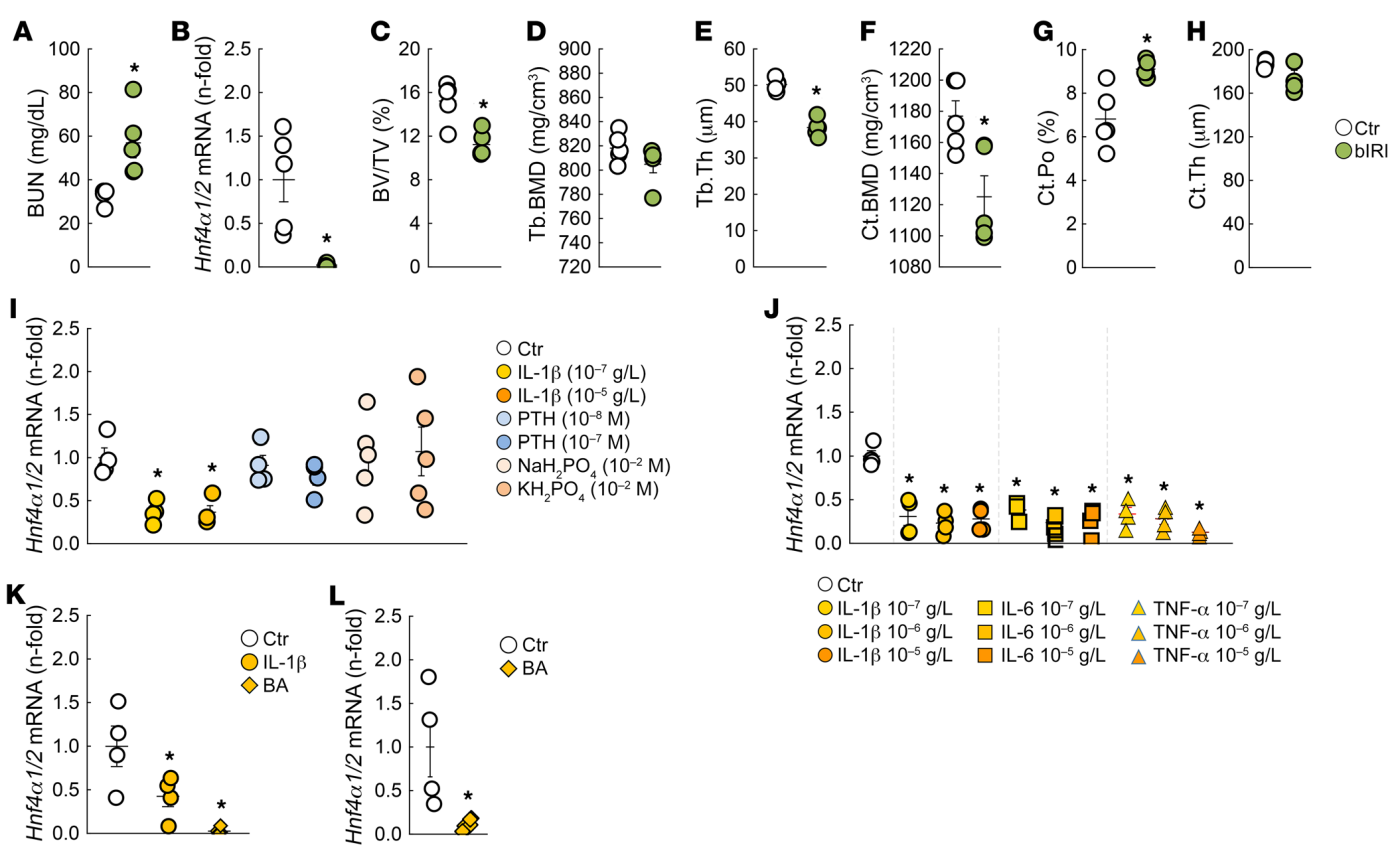
Bone *Hnf4α* expression is reduced in mice with CKD and in response to acute and chronic inflammation. (**A**) Renal function in 20-week-old sham-operated and bilateral ischemia/reperfusion injury (bIRI) WT male mice assessed by measurements of blood urea nitrogen (BUN). (**B**) Bone *Hnf4α* expression levels in 20-week-old sham and bIRI male mice. (**C**–**H**) Microtomography analysis of femur metaphysis secondary spongiosa (**C**–**E**) and femur cortical bone at metaphysis (**F**–**H**) in 20-week-old WT sham and bIRI mice. Ct, cortical; Po, porosity. Values are expressed as the mean ± SEM. *n* ≥ 4 per group; *P* < 0.05 versus *sham. (**I** and **J**) *Hnf4α* mRNA expression in a representative experiment performed at least 3 times in differentiated primary BMSC cultures isolated from WT mice treated for 6 hours with different concentrations of IL-1β, PTH, inorganic phosphate salts, IL-6, or TNF-α. (**K** and **L**) mRNA expression of bone *Hnf4α* in tibiae from WT mice injected with saline (Ctr), IL-1β, or heat-killed *Brucella abortus* (BA) 6 hours (**K**) or 14 days (**L**) after injection. Values are expressed as the mean ± SEM. *n* ≥ 4 per group; corrected *P* < 0.05 versus *Ctr. Statistical analysis was performed with an unpaired Student’s *t* test (**A**–**H** and **L**) or with an ANOVA followed by post hoc *t* tests and multiple-testing correction using the Holm-Bonferroni method (**I**–**K**).

*Hnf4α* expression is nearly completely suppressed in bone from patients and mice with CKD (Figure 1). A similar reduction is observed in a surgical bilateral ischemia/reperfusion injury (bIRI) model of acute kidney injury to CKD. At 20 weeks of age, 8 weeks after bIRI surgery, mice showed impaired kidney function paralleled by reduction in osseous *Hnf4α* and reduced trabecular and cortical bone mass compared with sham-operated mice (Figure 8, A–H). In CKD, low-grade inflammation, hyperparathyroidism, and hyperphosphatemia are among the major systemic disturbances that affect bone metabolism and structure (36–41). To determine whether these factors might also be responsible for the osteoblastic reductions in *Hnf4α*, we tested the effects of IL-1β, parathyroid hormone (PTH) and phosphate salts, NaH_2_PO_4_, and KH_2_PO_4_ in BMSCs cultured for 3 weeks in osteogenic medium. *Hnf4α* was reduced 6 hours after treatment with IL-1β but not in response to PTH and phosphate (Figure 8I), suggesting that inflammation might be responsible for *Hnf4α* suppression in CKD. Since the effects of inflammation on bone depend on the specific cytokines involved (42), we further tested the effects of 3 major cytokines in the same model, IL-1β, IL-6, and TNF-α, and found that all 3 cytokines similarly reduced *Hnf4α* expression in culture. We next used 2 in vivo models of inflammation: the *Brucella abortus* (BA) mouse model (43) that develops acute and chronic inflammation starting at 3 hours and lasting through 14 days after a single intraperitoneal injection of heat-killed bacteria (44, 45), and IL-1β administration (45, 46). Six hours after a single injection of IL-1β or BA, *Hnf4α* osseous expression was reduced by at least 50%, and it remained low 14 days after BA administration (Figure 8, K and L). These results suggest that inflammation is a powerful inhibitor of *Hnf4α* that might contribute at least in part to its suppression in ROD (Figure 8J).

### Genetic overexpression of Hnf4α2 in osteoblasts corrects bone alterations in mice with ROD.

**Figure 9:**
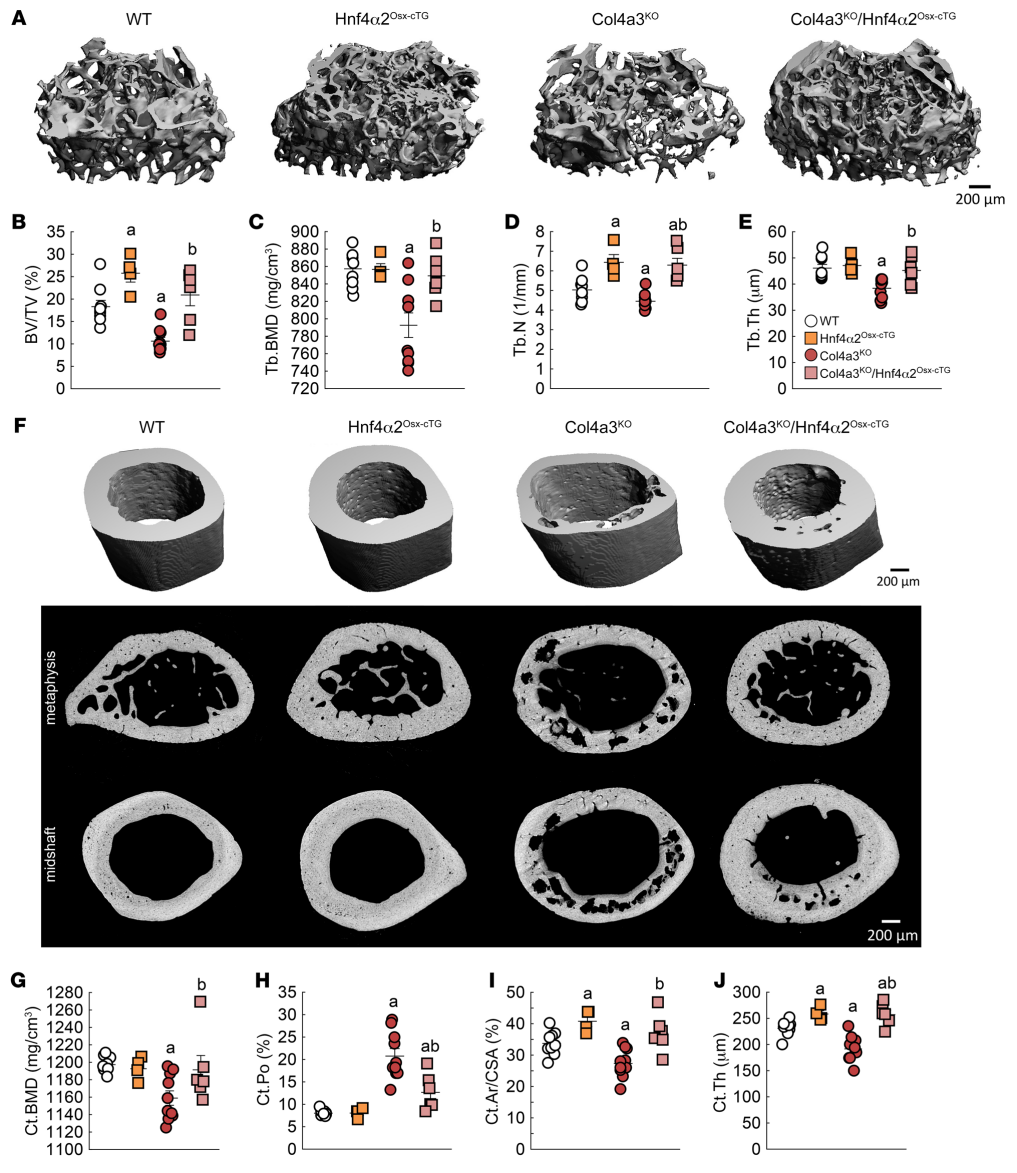
Genetic overexpression of *Hnf4α2* in osteoblasts prevents bone loss in mice with CKD. Microtomography analysis of femur metaphysis secondary spongiosa (**A**–**E**) and femur cortical bone at metaphysis (**F**, middle) and at midshaft (**F**, top and bottom, and **G**–**J**) in 20-week-old WT, Hnf4α^Osx-cTG^, Col4a3^KO^, and Col4a3^KO^/Hnf4α^Osx-cTG^ mice. Ar, area; CSA, cross-sectional area. Values are expressed as the mean ± SEM. *n* ≥ 8 per group; *P* < 0.05 versus ^a^WT, ^b^Col4a3^KO^. Statistical analysis was performed with an ANOVA followed by post hoc *t* tests to determine statistical differences and multiple-testing correction using the Holm-Bonferroni method.

Since HNF4α2 mediates the major osteogenic functions of HNF4α in osteoblasts, we created mice overexpressing *Hnf4α2* specifically in osteoblasts (Hnf4α2^Oc-cTG^) or pre-osteoblasts (Hnf4α2^Osx-cTG^) and determined the impact of increased *Hnf4α2* expression on the development of ROD in mice. Mirroring previous results on osteoblast-specific deletion of *Hnf4α* (Figure 5 and Supplemental Figure 2), Hnf4α2^Oc-cTG^ male mice showed increased trabecular bone mass at 12 weeks, but no effect on cortical bone envelope (Supplemental Figure 7, A–J). Overexpression of *Hnf4α2* earlier in the osteoblastic lineage led to an increase in trabecular and cortical bone mass in Hnf4α2^Osx-cTG^ animals compared with WT male littermates (Supplemental Figure 7, K–T), also consistent with the effects of *Hnf4α* deletion in pre-osteoblasts (Supplemental Figure 4) and demonstrating that HNF4α2 is a major early determinant of bone mass. To restore *Hnf4α2* expression in mice with CKD, we crossed Hnf4α2^Osx-cTG^ mice to Col4a3^KO^ mice. As previously shown (33), at 20 weeks of age, Col4a3^KO^ male mice showed trabecular bone loss compared with WT mice (Figure 9, A–E). Overexpression of *Hnf4α2* in Col4a3^KO^ mice prevented this bone loss, and compound Col4a3^KO^/Hnf4α2^Osx-cTG^ male mice displayed higher trabecular bone volume, bone mineral density, number, and thickness compared with Col4a3^KO^ mice (Figure 9, A–E). In addition to trabecular bone loss, Col4a3^KO^ male mice also showed reduced cortical bone mass, and increased cortical bone porosity (Figure 9, F–J), a distinct feature of ROD. Overexpression of *Hnf4α2* in Col4a3^KO^ mice reduced the number of pores and increased cortical bone mineral density, bone volume, and thickness (Figure 9, F–J). We detected similar effects in female mice (Supplemental Figure 8), and together, this suggests that *Hnf4α2* deficiency plays a major role in the pathogenesis of ROD, and that correction of *Hnf4α2* might prevent onset and progression of ROD.

## Discussion

Despite major advances in prevention and treatment of CKD, the pathology of ROD remains poorly understood, and effective strategies for its treatment, designed to improve bone health and prevent fractures, are lacking. Fracture incidence of the appendicular skeleton more than doubled from 1992 to 2009 in patients with CKD grade 5 requiring dialysis (47), and health care–associated costs after fracture in patients with CKD exceeded $600 million in 2010 (48). Anabolic and antiresorptive agents currently used in the treatment of osteoporosis underperform in the setting of ROD, suggesting that ROD is a more complex bone disease and that many critical parameters pertaining to intrinsic cellular alterations may play a major role.

We identified hepatocyte nuclear factor 4α (HNF4α) as a bone transcription factor that stimulates osteoblastogenesis and osteogenesis. We have shown that *Hnf4α* mRNA is expressed in cultured MC3T3-E1 pre-osteoblasts, primary osteoblasts, and mouse bone extracts. The longest isoform, *Hnf4α2*, which represents about 50% of total *Hnf4α*, shows the highest osteogenic potential. Cells overexpressing *Hnf4α2* showed increased expression of markers of osteoblastic differentiation. This suggests that HNF4α2 increases osteoblastic recruitment and accelerates differentiation. In contrast, cell cultures from mice lacking *Hnf4α* showed decreased expression of osteogenic markers compared with WT cells. In vivo, osteoblast-specific deletion of *Hnf4α* in healthy mice results in osteopenia. This suggests that *Hnf4α* expression is essential in maintaining bone mass. Importantly, we have also found that osseous HNF4α expression is reduced in patients and animals with CKD, and that overexpression of *Hnf4α2*, in pre- and mature osteoblasts, reduced skeletal abnormalities in animals with CKD. Taken together, our data indicate that HNF4α is a major transcriptional regulator of osteoblast metabolism and osteogenesis. These data suggest a role as a potential therapeutic target, as well as a prognostic marker, for ROD.

HNF4α belongs to the nuclear receptor (NR) family and has been characterized as a transcription factor with a restricted pattern of expression limited to liver and a few other endodermal organs, including kidney and pancreas, since it was identified in the early 90s (49, 50). HNF4α has been associated with the transcriptional regulation of liver morphogenesis and thought to be involved in defining hepatocellular identity (51). However, compared with HNF1α and HNF6, HNF4α has been found to be a widely acting transcription factor, at least in liver and pancreas, consistent with the observation that it is an unusually abundant and constitutively active transcription factor (52). Consistent with these findings, HNF4α expression has been documented later in large amounts in liver, stomach, small intestines, colon, pancreas, and kidney (21) and at lower levels in testis, ovary, lung, spleen, and skin (30, 53). Thus, far from being restricted to one tissue, HNF4α appears to be widely distributed and functional. Given its wide distribution, HNF4α mutations are associated with a wide spectrum of diseases (24–27, 54–56). Prior to our study, a single report has found that HNF4α might be a central regulator of genes also involved in osteoporosis (29).

In osteoblasts, HNF4α regulates canonical osteogenic genes, such as *Alpl*, *Sp7*, and *Runx2*, several integrins (*Itga2*, *Itga2b*, *Itgb3*, *Itgb8*, *Itga5*), and β-catenin signaling genes, including *Tcf5* and *Tcf7*. We show that HNF4α regulates canonical osteogenic genes, and only one previous study has shown the ability of HNF4α to bind *Dmp1* promoter, albeit in the pancreas (52). While HNF4α regulates purely osseous genes, the relationship between HNF4α and β-catenin appears to be a reciprocal negative regulatory loop (57), suggesting that the osteogenic activity of HNF4α might be tempered by its antagonistic effects on β-catenin signaling. A hallmark of HNF4α activity in osteoblasts resides in its capacity to bind and regulate genes coregulated by cAMP/PKA/CREB pathways. Several studies have shown that PKA inhibits HNF4α (58) and that interactions with CREB-binding protein modulate HNF4α transcriptional activity (59, 60). The finding that HNF4α regulates cAMP/PKA/CREB pathways indicates that a metabolic loop might link these two signals.

As shown in prior studies (61), HNF4α appears to also regulate cell death pathways in osteoblasts, and these pathways are exacerbated by increased proinflammatory signaling when HNF4α is mutated (62). Interestingly, we also found a persistent proinflammatory signature in the bones of Hnf4α^Oc-cKO^ mice, but no alterations in proinflammatory signaling in cultured osteoblasts overexpressing *Hnf4α*, suggesting that reduced *Hnf4α* is a gateway to increased bone inflammation and that HNF4α is downstream of inflammatory stimuli. Finally, HNF4α activity in osteoblasts also largely regulates known HNF4α-responsive genes involved in glycolytic and lipid metabolism as well as cell response to xenobiotics, similar to observed functions of HNF4α in other organs (63, 64), suggesting a “conserved” function for HNF4α, irrespective of the organ. Taken together, these results suggest that impaired osseous HNF4α signaling affects bone structure and metabolism as a result of modifications of both “conserved” and “organ-specific” HNF4α functions.

HNF4α is expressed as multiple isoforms with varying transcriptional regulatory potentials, and currently 12 distinct HNF4α proteins have been annotated in both humans and mice (30, 31). HNF4A isoforms are generated by 2 alternative promoters, P1 and P2, which result in over 60 potential HNF4A homo- or heterodimer isoforms with different impacts on gene expression regulation (30). Studies often do not distinguish between isoforms, and refer collectively to “HNF4α,” as the different isoforms are assumed to be functionally equivalent given the conservation of functional domains. The different isoforms differ only at N- and C-termini, which are responsible for activating and repressing transcription. In our studies, we identified 2 osteoblast-expressed HNF4α isoforms, α1 and α2. HNF4α2 is expressed at the same levels as HNF4α1 in bone and only minimally differs from HNF4α1 by 10 amino acids in the repressor region. However, we further found that mainly HNF4α2 regulates the expression of major metabolic and osteogenic genes in osteoblasts.

In all vertebrates, there are fundamental structural and metabolic differences between the trabecular bone, defined by a larger remodeling area and higher turnover rate, and the dense and less metabolically active cortical bone (65–69). Consequently, deletion of *Hnf4α* in mature osteoblasts and osteocytes mostly affected trabecular bone at peak bone mass in 12-week-old mice. Following deletion or overexpression of *Hnf4α* earlier in the osteoblastic lineage using Osterix-Cre, *Hnf4α* also affected the cortical architecture in both male and female mice, suggesting that recruitment of a larger pool of cells is sufficient to alter both cancellous and cortical bone envelopes. Finally, despite a few subtle differences, deletion or overexpression of *Hnf4α* tends to affect bone metabolism and architecture in a similar manner, in both male and female mice. However, deletion of *Hnf4α* has a more profound effect on the female skeleton, as it affects both cortical and trabecular bone. Although beyond the scope of this study, this suggests that the number of cells affected by HNF4α2 absence might be larger than in the male skeleton. Alternatively, HNF4α2 might be responsible in part for the sexual dimorphism affecting the skeleton, consistent with the role of HNF4α as a core transcription factor involved in the expression of sexually dimorphic genes (70–72).

In addition, HNF4α2 regulates most of the impaired osteogenic and metabolic activities associated with reduced *Hnf4α* expression in patients and animals with ROD. We found that HNF4α2 is downregulated in the bone of patients with ROD, possibly owing to systemic inflammation, which also suppresses its expression in other tissues (73–75). Although no other studies have investigated the expression of HNF4α in other organs in patients and animal models of CKD, one would expect reduced HNF4α expression to be a multiorgan determinant in CKD. Given the primary role of HNF4α in primary tubular cell repair and function (76–78), it is possible that a decline in renal HNF4α expression also occurs as CKD progresses. Nonetheless, overexpression of *Hnf4α2* in osteoblasts was sufficient to correct in large part the skeletal deformities in mice with CKD, suggesting that activating osteoblastic HNF4α2 could open novel therapeutic opportunities for a myriad of metabolic and skeletal disorders.

### Limitations.

HNF4α has been the continuous focus of studies investigating its role in the control of cell identity and regulation of cellular metabolism in endodermal organs. We have established the major role of HNF4α2 in osteoblasts as a major regulator of osteogenesis, with special emphasis on ROD. However, we have not tested the specific effect of HNF4α1 on osteoblasts and the skeleton in healthy mice or animals with CKD, nor have we explored the indirect metabolic impact of HNF4α2 at a systemic level. Further explorations of both isoforms will help elucidate the full biological activities of this molecule.

## Methods

### Human bone biopsies.

Iliac crest bone biopsies from 9 healthy volunteers and 20 patients with CKD were obtained using an 11 G × 10 cm bone marrow biopsy needle and immediately frozen for further analyses. The control samples were obtained from a bone tissue bank from the Hospital das Clínicas da Faculdade de Medicina da USP (São Paulo, Brazil). The tissue came from post-traumatic deceased donors, from whom several organs (including bone) were flash-frozen immediately postmortem and distributed to different recipients and researchers. Biopsies and RNA extraction were performed similarly in control and all other experimental samples. A second specimen was obtained from patients with CKD using a 7 mm Bordier trephine, after double labeling with tetracycline (20 mg/kg/d) for bone histomorphometry measurements and segregation in low (9 biopsies) or high (11 biopsies) bone remodeling groups. In all groups, a balanced number of male and female participants were used (Supplemental Table 1).

### Animals.

Hnf4α^fl/+^ [B6.129X1(FVB)-Hnf4αtm1.1Gonz/J], Col4a3^+/–^ (129-Col4a3tm1Dec/J), osteocalcin-Cre [B6.FVB-Tg(BGLAP-cre)1Clem/J], and Osterix-Cre [B6.Cg-Tg(Sp7-tTA,tetO-EGFP/cre)1Amc/J] mice were purchased from The Jackson Laboratory. The Hnf4α2^STOP/+^ mice were created at Northwestern University by the transgenic mouse facility. They harbor a targeted mutation of the Gt(ROSA)26Sor locus with a *loxP*-flanked STOP cassette preventing transcription of an *Hnf4α2* transgene. *Hnf4α2* is expressed following Cre-mediated excision of the STOP cassette. Hnf4α^fl/fl^ and Hnf4α2^STOP/STOP^ mice were respectively crossed to generate Hnf4α^fl/fl^ and Hnf4α^STOP/STOP^ (WT), Hnf4α^fl/fl^-Cre (Hnf4α^cKO^), and Hnf4α^STOP/STOP^-Cre (Hnf4α2^cTg^). Hnf4α^STOP/STOP^-Cre mice were further crossed with Col4a3^+/–^ mice to generate Col4a3^+/+^ Hnf4α^STOP/STOP^ (WT), Col4a3^+/+^ Hnf4α^STOP/STOP^-Cre (Hnf4α2^cTg^), Col4a3^–/–^ Hnf4α^STOP/STOP^ (Col4a3^KO^), and Col4a3^–/–^ Hnf4α^STOP/STOP^-Cre (Col4a3^KO^/Hnf4α2^cTg^). In all cohorts, we further backcrossed the F_1_ heterozygotes to generate incipient congenic strains that contained 94% C57BL/6J genome and maintained the newly created strains separately for more than 5 generations as in prior studies (33, 34). All mice were kept in our vivarium on a standard control diet. Each mouse was genotyped twice, at weaning and after sacrifice, using REDExtract-N-Amp Tissue PCR Kit (Sigma-Aldrich). For all studies, we report results obtained in male and female littermate mice. To test the in vivo effect of inflammation on bone *Hnf4α* expression, 6-week-old C57BL/6J male mice (The Jackson Laboratory) were injected intraperitoneally with mouse recombinant IL-1β, 50 ng/g of body weight (Sigma-Aldrich), heat-killed *Brucella abortus* strain 1119, 3.5 × 10^6^ particles (National Veterinary Services Laboratories), or saline control as previously described (45, 46). Mice were sacrificed after 6 hours or 14 days, and bones were collected to assess bone RNA isolation followed by reverse transcriptase PCR (RT-PCR).

### Bilateral ischemia/reperfusion injury.

Bilateral ischemia/reperfusion injury was performed in 12-week-old mice anesthetized with xylazine (10 mg/kg i.p.) and ketamine (90–120 mg/kg i.p.). Briefly, after a small midline abdominal incision, both renal pedicles were occluded with a microaneurysm clamp. The abdomen was partially closed, temporarily, with sutures, and body temperature was monitored by rectal probe and controlled with a heating pad. After 23 minutes, the clamp was removed, and reperfusion was visually confirmed. The abdomen was closed with a 6-0 suture, and the skin was closed with Michel miniature clips. Mice were maintained on a thermostatically controlled warm plate at 37°C during and after surgery.

### RNA isolation, RT-PCR, and RNA-Seq.

We isolated total RNA from tissues and from cell cultures using TRI Reagent (MRC) and purified RNA using an RNeasy kit (Qiagen).

For RNA-Seq, the total RNA library for each individual sample was prepared using the TruSeq Total RNA-Seq Library Preparation Kit (Illumina), and the barcoded cDNA libraries were sequenced for 100 bp single reads using Illumina NextSeq to generate 30 to 40 million reads per sample. Reads from each library were mapped to the human or mouse transcriptome and genome and filtered using StrandNGS software suite (Strand Life Sciences), following Strand alignment and filtering pipelines. Reads were normalized using DESeq, and we used baseline transformation to the median for each sample. Fold change and *P* value were calculated using moderated 2-tailed *t* test, and data were used for subsequent downstream pathway analyses using the Ingenuity Pathway Analysis platform (IPA, QIAGEN).

For RT-PCR, we synthesized first-strand cDNA (iScript cDNA Synthesis Kit, Bio-Rad Laboratories) and used the iCycler iQ real-time PCR detection system, iQ SYBR Green Supermix (Bio-Rad Laboratories), and adequate primer pairs for real-time quantitative PCR analysis. Primer sequences are provided in Supplemental Table 3. The threshold of detection of each gene expression was set at optimal reaction efficiency. The expression was plotted against a standard dilution curve of relative concentration, normalized to 60S ribosomal protein L19 (Rpl19) expression in the same sample, and expressed as fold change versus respective controls.

### Hnf4α isoform assessment.

Reference sequences corresponding to the 12 annotated *Hnf4α* isoforms were identified from the NCBI database (http://www.ncbi.nlm.nih.gov) using a similar previously reported strategy (32). Primer sequences are provided in Supplemental Table 4.

### Chromatin immunoprecipitation, sequencing, and peak calling.

We first immunoprecipitated HNF4α in samples isolated from cells overexpressing *Hnf4α1*, *Hnf4α2*, or Ctr MC3T3 cultures using 2 different polyclonal anti-HNF4α antibodies purchased from Aviva Systems Biology and Abcam, respectively. In parallel, we generated 2 stable cell lines overexpressing *Hnf4α2* coupled with a carboxy-terminal (Hnf4α2^C-Halo-Tg^) or amino-terminal (Hnf4α2^N-Halo-Tg^) Halo tag and used an anti-Halo antibody to immunoprecipitate these cells. Cells were cultured into osteoblastic medium for 21 days. Cell cultures were subjected to ChIP assay following the protocol provided by the manufacturer (SimpleChIP Plus Enzymatic Chromatin IP Kit with magnetic beads, Cell Signaling Technology). Briefly, protein-chromatin cross-linking was carried out in cell medium containing 1% formaldehyde (Sigma-Aldrich) at room temperature for 10 minutes. After the cross-linking reaction was stopped using 10× glycine for 5 minutes, cells were washed 3 times in PBS and scraped into cold PBS containing protease inhibitor cocktail. Collected cells were centrifuged at 2,000*g* for 5 minutes at 4°C. After nuclei extraction, chromatin was digested with micrococcal nuclease, and nuclear membranes were disrupted by sonication. Lysates were clarified by centrifugation at 9,400*g* for 10 minutes at 4°C. For immunoprecipitation (IP), 10 μg digested, cross-linked chromatin per reaction was incubated with anti-HNF4α antibodies: 5 μg OASG03561 (RRID:AB_2895200; Aviva Systems Biology), 10 μg ab41898 (RRID:AB_732976; Abcam), or 10 μg anti–Halo tag antibody (G9281, RRID:AB_713650; Promega) for 4 hours at 4°C. Then, 30 μL Dynabeads Protein G Magnetic Beads was added to the IP chromatin solution and incubated for 2 hours at 4°C. After several washing steps using buffers with ascending NaCl concentrations, chromatin was eluted, and the supernatant was incubated with proteinase K overnight at 65°C to reverse cross-linking. Finally, DNA fragments were purified using silica columns. For each condition, we used 3 separate biological replicates and 1 input control. The total DNA library for each individual sample was prepared using the TruSeq ChIP-Seq Library Prep Kit (Illumina), and the barcoded cDNA libraries were sequenced for 100 bp single reads using the Illumina HiSeq 4000. The ENCODE pipeline (https://github.com/ENCODE-DCC/chip-seq-pipeline2) v1.7.1 was used to identify naive overlapping peaks in each experiment. Enriched regions were consolidated based on their representation in 2 or more experiments as follows: HNF4α1/2 peaks if detected in samples expressing Hnf4α1 and Hnf4α2; HNF4α1 peaks if detected in 2 or more samples overexpressing Hnf4α1 or Ctr cells; HNF4α2 peaks if detected in 2 or more samples overexpressing Hnf4α2 or Ctr cells.

### Cell culture.

We stably transfected murine osteoblast-like cell line MC3T3-E1 subclone 4 (ATCC CRL-2593) using the 4D-Nucleofector System (Lonza) to generate cells that overexpressed *Hnf4α1*, *Hnf4α2*, or an empty vector (Ctr). Primary bone marrow stromal cells (BMSCs) and osteoblasts were collected from 8-week-old male mice. Bone marrow was isolated by centrifugation and primary osteoblasts by sequential trypsin/collagenase digestion of bones devoid of bone marrow. Cell lines and primary cells were cultured for 21 days in αMEM medium supplemented with 10% FBS (Corning), 10 U/mL penicillin, 100 μg/mL streptomycin (Thermo Fisher Scientific), 10 mM β-glycerophosphate, and 50 μg/mL ascorbic acid (Sigma-Aldrich) to induce differentiation (33, 45, 46). To identify the molecular mechanisms regulating *Hnf4α*, BMSCs were cultured for 21 days in osteogenic medium and then challenged with escalating doses of recombinant mouse cytokines (IL-1β, IL-6, and TNF-α, Cell Signaling Technology), recombinant PTH 1–34 (Sigma-Aldrich), and 10 mM inorganic phosphate salts (sodium phosphate monobasic or potassium phosphate monobasic, Sigma-Aldrich) for 6 hours.

### 3D microtomography.

We scanned ethanol-fixed whole femora at 6 μm isotropic voxel size with high-resolution microtomography (μCT50, Scanco Medical) at an energy level of 70 keV and intensity of 57 μA. The trabecular bone structure was analyzed within 1 mm of the secondary spongiosa of the distal femur underneath the growth plate. The cortical bone structure was analyzed within 1 mm at the midshaft of each femur. All grayscale images were segmented using a fixed Gaussian filter and threshold for all data. Representative segmented images were generated for the trabecular and cortical bone, as previously shown (33, 79–82).

### Histology and histomorphometry.

We injected mice with alizarin red S at 7 and 2 days before harvest for intravital staining of active mineralization fronts (33, 83). We measured femur and tail lengths using a slide caliper to evaluate bone growth (33, 83, 84). We fixed and dehydrated the femur samples in ethanol, and embedded them in methylmetacrylate (MMA) at low temperature. For histology analyses, we cut non-serial 5 μm MMA slices (Leica Microsystems Inc.) and captured bright-field and fluorescence microscopy images (Leica Microsystems Inc.). We analyzed unstained longitudinal femoral sections, modified Goldner Trichrome–stained sections, and sections stained for tartrate-resistant acid phosphatase (TRAcP) activity according to previously described methods (80).

### Metabolomics.

We isolated metabolite fractions from cell cultures by methanolic extraction. Briefly, cells were rinsed twice with ice-cold saline solution. Then, 1 mL of 80% methanol (vol/vol) was added to cells and incubated at 80°C for 20 minutes. Cells were extracted by scraping. Cell lysates were subjected to 3 consecutive freeze-thaw cycles and centrifuged at 20,000*g* for 15 minutes at 4°C. Metabolite-containing supernatants were dried, and then reconstituted in 50% acetonitrile (vol/vol) and centrifuged at 20,000*g* for 15 minutes at 4°C. Supernatants were analyzed by high-performance liquid chromatography and high-resolution mass spectrometry and tandem mass spectrometry (HPLC-MS/MS). Data acquisition and analysis were performed using Xcalibur 4.1 and Tracefinder 4.1 software (Thermo Fisher Scientific).

### Next-generation sequencing data.

Next-generation sequencing data were deposited in the NCBI’s Gene Expression Omnibus (GEO) database as follows: transcriptomic analyses of bone biopsies from patients with ROD (GSE194056); overexpression of *Hnf4α1* and *Hnf4α2* in MC3T3 osteoblasts (GSE190315); ChIP-Seq of HNF4α in cells overexpressing HNF4α1 or HNF4α2 (GSE190314); bone RNA-Seq of WT and HNF4α^Oc-cKO^ mice (GSE190313).

### Statistics.

Data are presented as mean ± SEM. We used 1-way ANOVA followed by 2-tailed post hoc *t* tests to test statistical differences and multiple-testing correction using the Holm-Bonferroni method (85) (Statistica software, Statsoft). Differences were considered statistically significant at *P* values less than 0.05.

### Study approval.

Bone biopsy samples were collected from patients who participated in previously approved institutional studies (61597616.0.000.0068 and 64157017.6.0000.0068). All participants provided written informed consent to have their samples stored for future use. The institutional ethics committee of the Hospital das Clínicas da Faculdade de Medicina da USP (HCFMUSP), Universidade de São Paulo, São Paulo, Brazil (664/97) approved this project. All animal studies in the present work were approved by the IACUC of Northwestern University.

## Author contributions

VD designed the study. All authors contributed to data acquisition and/or data interpretation. LMDR, CSWM, and RMAM performed the biopsies and provided the RNA. MMC, GC, BHT, CF, JS, and XW performed the experiments and acquired and interpreted the data. IBS, HM, TLN, and RMAM provided clinical input and critical interpretation. MMC, AM, and VD drafted the manuscript. All authors revised the manuscript. All authors reviewed and approved the final version of the manuscript.

## Supplementary Material

Supplemental data

## Version 1 04/20/2023

In-Press Preview

## Version 2 06/01/2023

Electronic publication
