# Supplementary material for: Transcription factor HNF4α2 promotes osteogenesis and prevents bone abnormalities in mice with renal osteodystrophy
Source: J Clin Invest. 2023 Jun 1;133(11):e159928. doi: 10.1172/JCI159928 (PMC10231994; doi:10.1172/JCI159928)
Supplement: Supplemental data [file jci-133-159928-s134.pdf]

Supplementary figures

Fig. S1

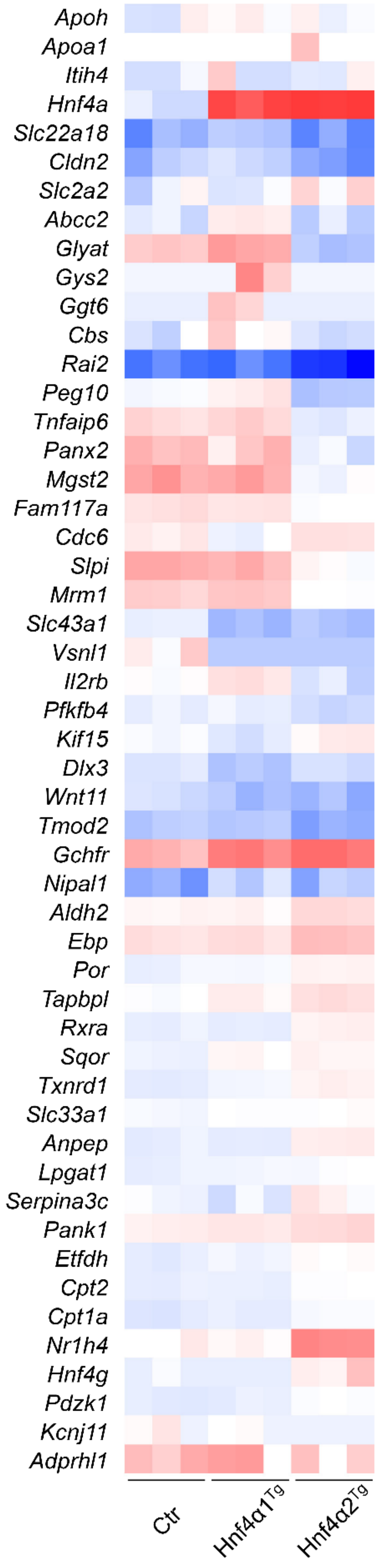

Fig. S1: HNF4α overexpression alters the expression of known HNF4α targets in osteoblasts. Heatmap-represented log-normalized expression of genes identified in Ctr, Hnf4α1^{Tg}, and Hnf4α2^{Tg} cultured MC3T3-E1 osteoblasts. Statistical tests were performed using an ANOVA test, followed by unpaired student's t test and corrected by the False Discovery Rate.

Fig. S2

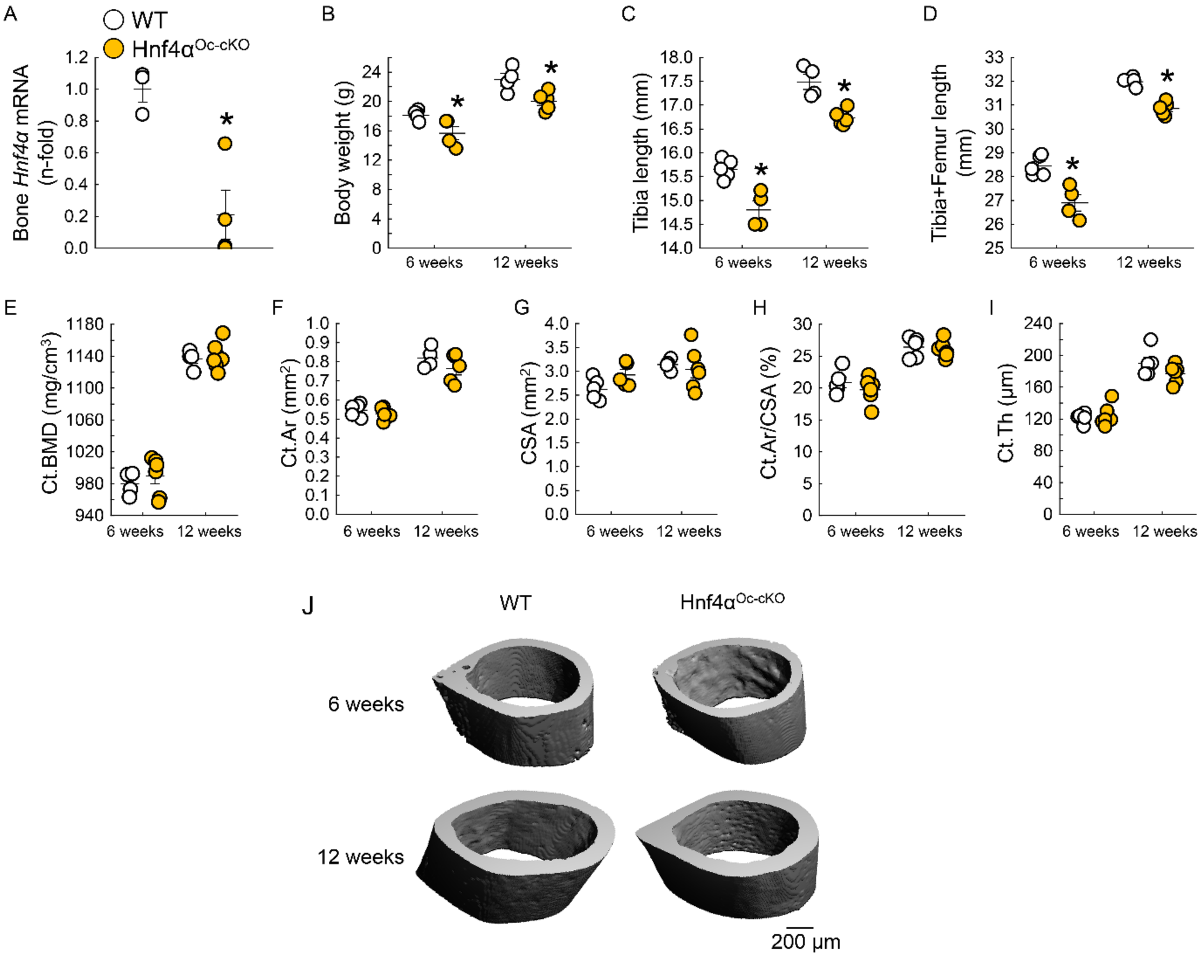

Fig. S2: Osteoblast-specific *Hnf4α* deletion leads to impaired bone growth. (A) Bone *Hnf4α* mRNA expression in femoral bone of adult (12 weeks) WT (n=3) and Hnf4α$^{Oc\text{-}cKO}$ (n=4) male mice, (B) body weight, (C-D) tibia and femur lengths, and (E-J) 3D microtomography of femur cortical bone analyzed at midshaft in young (6 weeks) and adult (12 weeks) WT and Hnf4α$^{Oc\text{-}cKO}$ male mice. Values are expressed as mean±SE. n≥5, $p<0.05$ vs. *age-matched WT. Statistical tests were performed using unpaired student's t tests.

Fig. S3

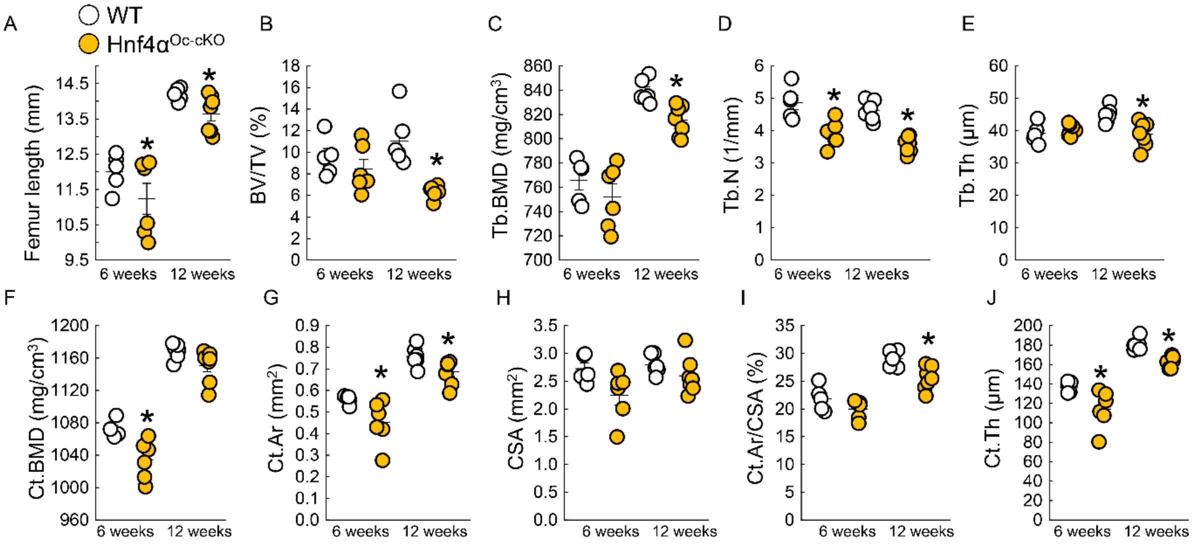

Fig. S3: Osteoblast-specific *Hnf4α* deletion leads to trabecular bone loss in female mice. (A) Femur length and (B-J) 3D microtomography of femur trabecular (B-E) and cortical (F-J) bone analyzed at midshaft in young (6 weeks) and adult (12 weeks) WT and Hnf4α$^{Oc\text{-}cKO}$ female mice. Values are expressed as mean±SE. n≥4 per group. $p<0.05$ vs. *age-matched WT. Statistical tests were performed using unpaired student's t tests.

Fig. S4

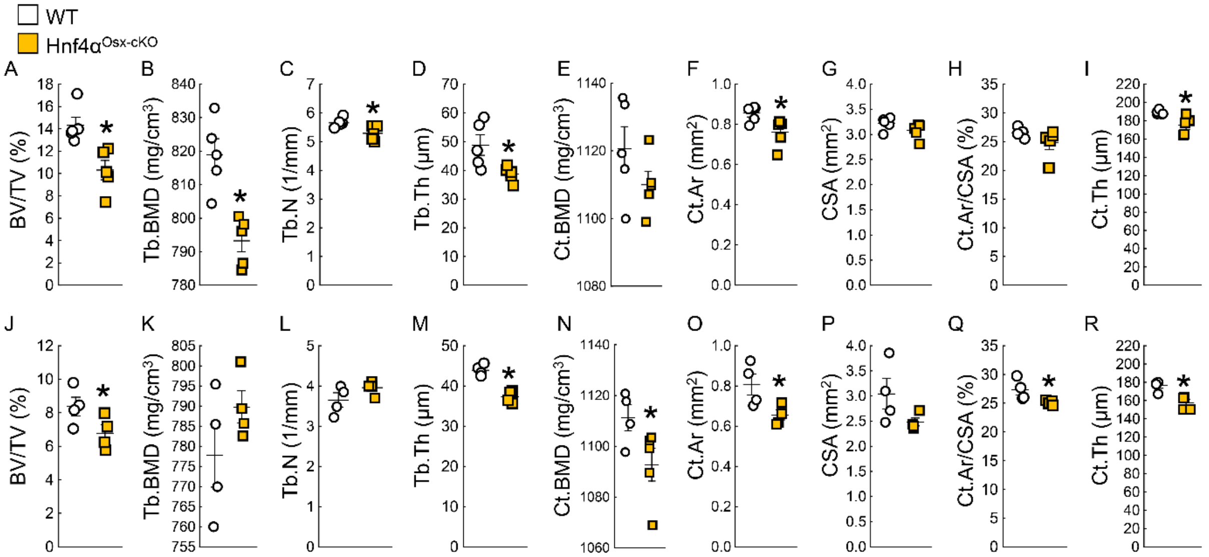

Fig. S4: Pre-osteoblast-specific *Hnf4α* deletion leads to trabecular and cortical bone loss in male and female mice. (A-R) 3D microtomography of femur trabecular (A-D and J-M) and cortical (E-I and N-R) bone analyzed at midshaft in adult (12 weeks) WT and Hnf4α$^{Oc\text{-}cKO}$ male (A-I) and female (J-R) mice. Values are expressed as mean±SE. n≥4 per group. $p<0.05$ vs. *sex and age-matched WT. Statistical tests were performed using unpaired student's t tests.

Fig. S5

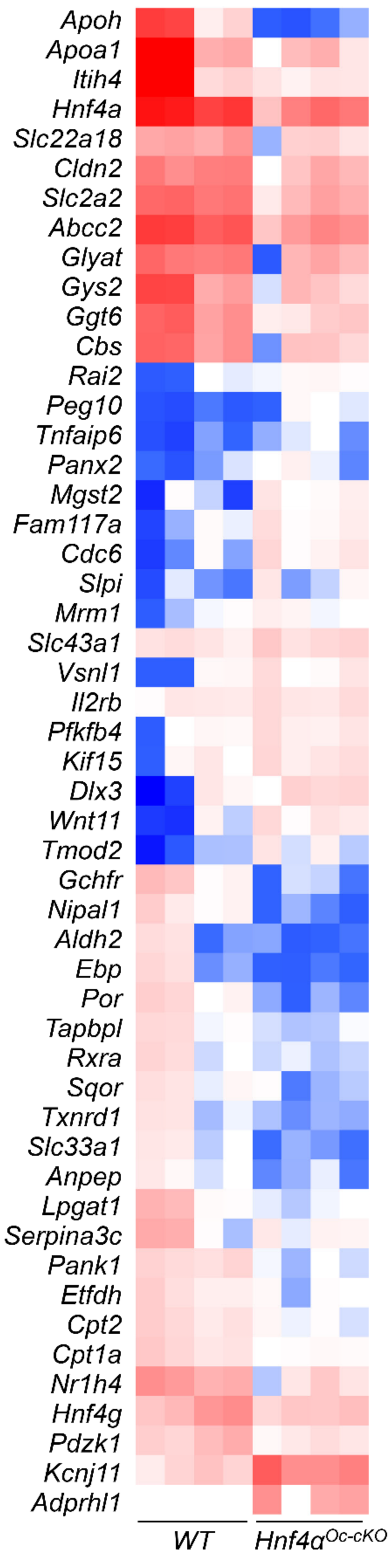

Fig. S5: Osteoblast-specific deletion of *Hnf4α* alters the expression of known HNF4α targets in bone. Heatmap-represented of log-normalized expression of known HNF4α regulated genes identified in WT and Hnf4α$^{Oc\text{-}cKO}$ mice. Statistical tests were performed using unpaired student's t test and corrected by the False Discovery Rate. Corrected $p<.05$, n=4 per group.

Fig. S6

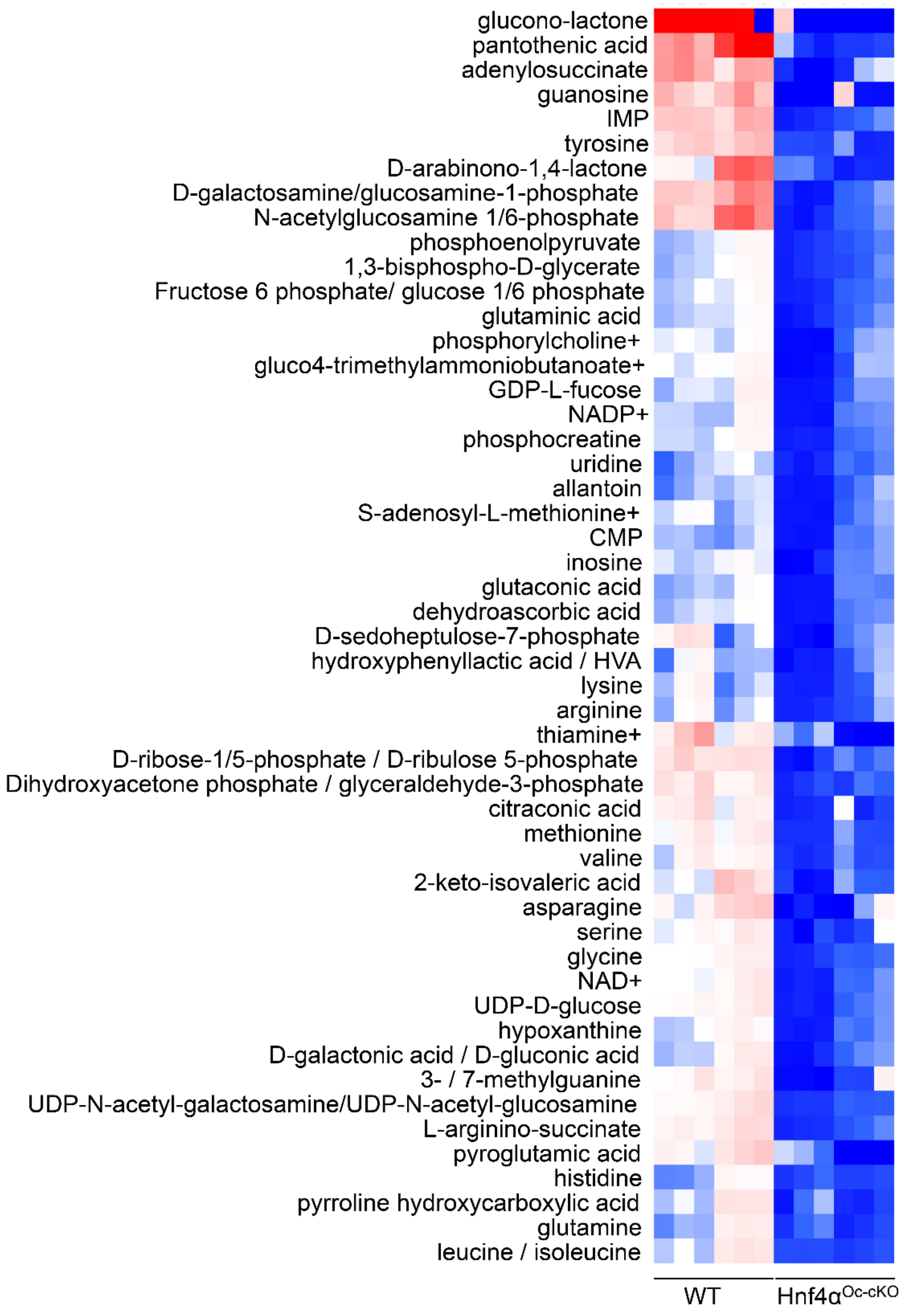

Fig. S6: Osteoblast-specific deletion of *Hnf4α* alters cell metabolism. Heatmap-represented concentration of differentially regulated metabolites identified by metabolomics analysis of WT and Hnf4α$^{Oc\text{-}cKO}$ cultured primary osteoblasts. Statistical tests were performed using unpaired student's t test and corrected by the False Discovery Rate. Corrected $p<.05$, $n=6$ per group.

Fig. S7

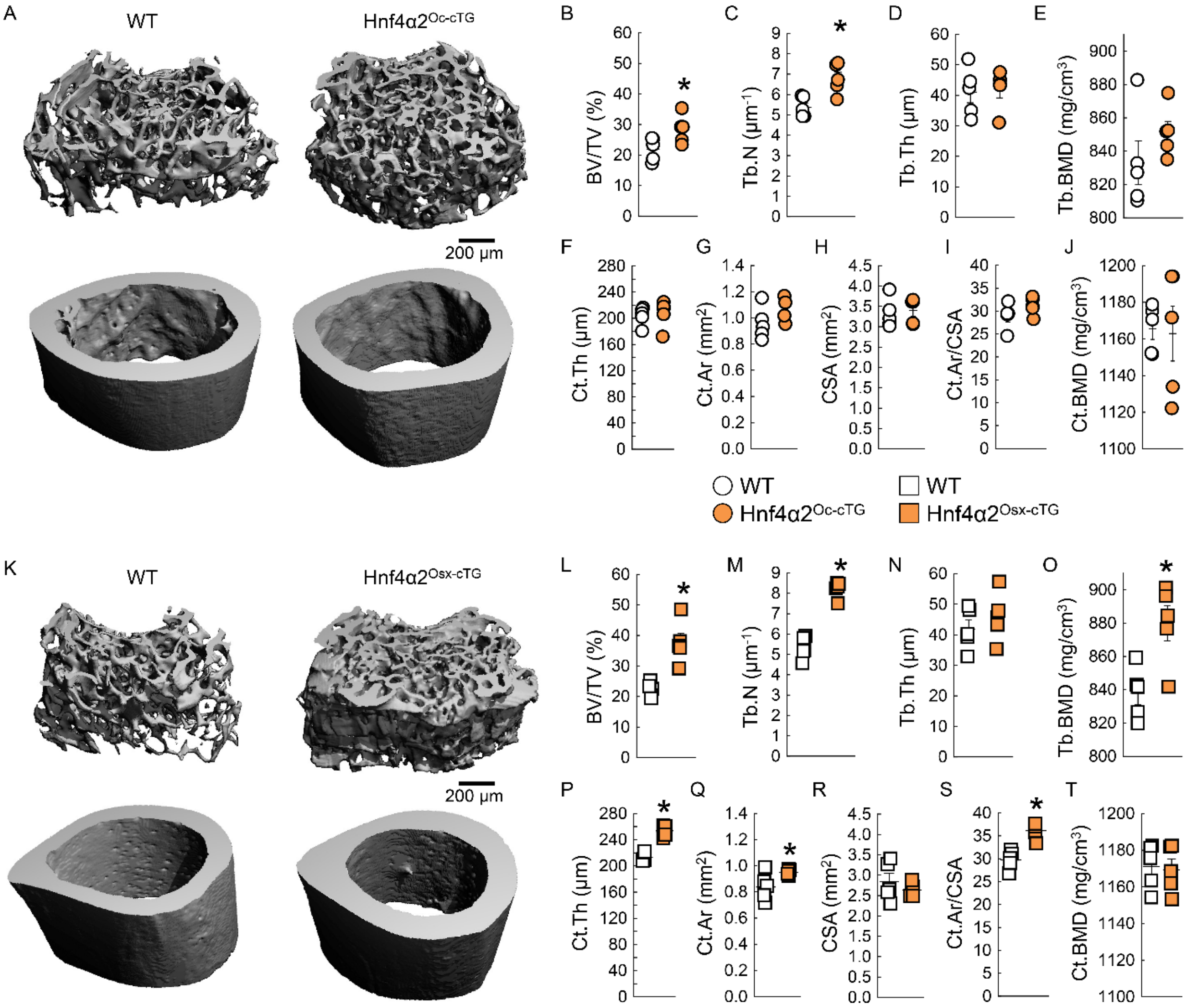

Fig. S7: *Hnf4α2* expressed in early differentiated osteoblasts stimulates trabecular and cortical osteogenesis. 3D microtomography analysis of femur metaphysis secondary spongiosa and midshaft cortical bone in 12 week-old (**A-J**) WT and Hnf4α$^{Oc\text{-}cTG}$ mice, and (**K-T**) WT and Hnf4α$^{Osx\text{-}cTG}$ male mice. (**B-E**, **L-O**) Quantitative analysis of trabecular bone parameters, and (**F-J**, **P-T**) quantitative analysis of cortical bone parameters. Values are expressed as mean±SE. n≥5 per group. $p<0.05$ vs. *WT. Statistical tests were performed using unpaired student's t tests.

Fig. S8

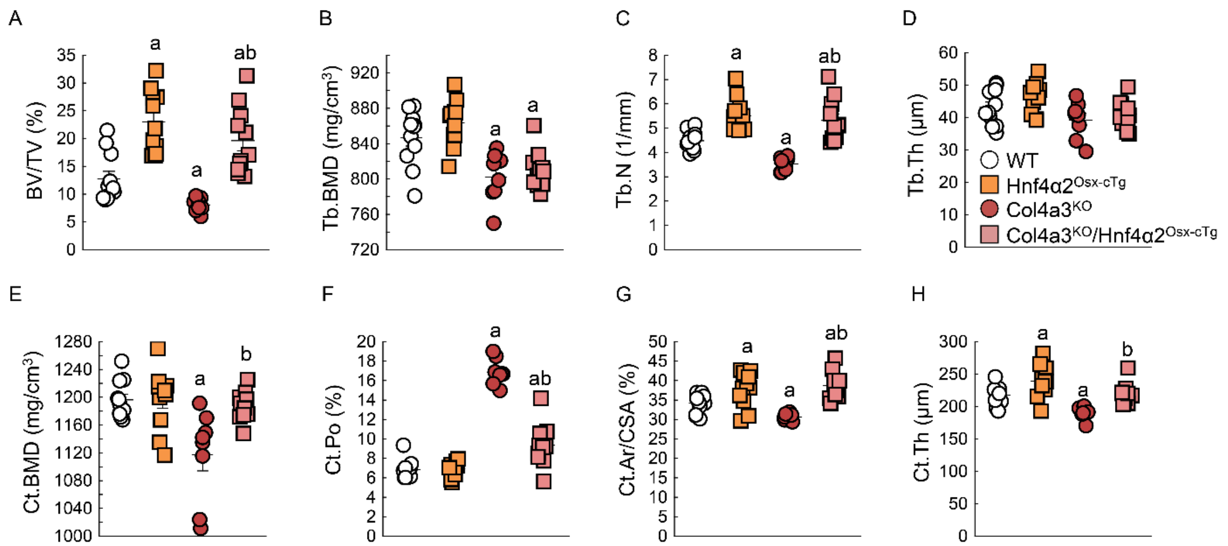

Fig. S8: Genetic overexpression of *Hnf4α2* in osteoblasts prevents bone loss in female mice with CKD. Microtomography analysis of (A-D) femur metaphysis secondary spongiosa, (E-H) femur cortical bone at metaphysis in 20 week-old WT, Hnf4α$^{Osx\text{-}cTG}$, Col4a3^{KO} and Col4a3^{KO}/Hnf4α$^{Osx\text{-}cTG}$ female mice. Values are expressed as mean±SE. n≥6 per group. Corrected $p<0.05$ vs. ^{a}WT, ^{b}Col4a3^{KO}. Statistical tests were performed using an ANOVA test followed by post-hoc t tests to test statistical differences and multiple testing correction using Holm-Bonferroni method.

Supplementary tables

Table S1. Main Demographic and Clinical Characteristics of the Patients Included in the Analysis.

Characteristic	Group			p value
	Healthy	LR-ROD	HR-ROD	
Patients, n(%)	9(31)	9(31)	11(38)	
Age (years), median (IQR)	47(43.49)	39(35.47)	39(32.54)	ns
Gender, female, n(%)	4(44.4)	4(44.4)	2(18.2)	ns
Race, non-white, n (%)	2(7)	4(14)	2(7)	ns
Weight, kg (IQR)	84(75,90)	56(50,67)	63(56,77)	0.00144257414
Height, meters (IQR)	1.70(1.65,1.75)	1.55(1.55,1.69)	1.67(1.50,1.74)	ns
Clinical Protocol				
Before PTX	-	0	5	
Before KTx	-	7	0	
OP Therapy	-	2	6	

PTX = parathyroidectomy; KTx = Kidney transplantation; OP = osteoporosis. Statistical tests were performed using an ANOVA test.

Table S2. Bone histomorphometry Analyses.

Age	6 weeks				12 weeks			
Sex	Males		Females		Males		Females	
Genotype<br><br>Parameter	**WT**	**Hnf4α$^{Oc\text{-}cKO}$**	**WT**	**Hnf4α$^{Oc\text{-}cKO}$**	**WT**	**Hnf4α$^{Oc\text{-}cKO}$**	**WT**	**Hnf4α$^{Oc\text{-}cKO}$**
O.Th (μm)	2.9±0.2	2.7±0.1	3.4±0.1	2.5±0.2*	2.6±0.1	2.2±0.1*	2.6±0.1	2.4±0.1
OS/BS (%)	22.4±2.1	7.2±1.7*	22.7±2.9	13.5±1.6*	11.4±4.1	3.9±1.1	11.8±0.8	8.2±0.9*
MAR (μm/d)	3.1±0.3	1.4±0.4*	2.6±0.1	1.5±0.2*	1.3±0.1	1.1±0.2	1.5±0.1	1.1±0.1*
BFR/BS(μm^3/μm^2/d)	1.5±0.1	0.9±0.2*	1.4±0.1	0.7±0.1*	0.5±0.1	0.4±0.2	0.8±0.1	0.6±0.1*
Oc.N/B.Pm(mm^{-1})	12.0±0.6	15.6±1.8	10.4±0.6	8.5±0.2	5.8±1.0	11.8±1.7*	6.9±0.2	6.7±0.8
OcS/BS (%)	14.7±0.9	19.9±1.2*	20.5±0.9	28.4±3.4	10.9±0.7	15.1±1.3*	12.3±1.1	14.6±2.2

O.Th = osteoid thickness; OS/BS = osteoid surfaces per bone surfaces; MAR = mineral apposition rate; BFR/BS=bone formation rate per bone surfaces; Oc.N/B.Pm.=number of osteoclasts per bone perimeter; Oc.S/BS=osteoclast surfaces per bone surfaces. Values are expressed as mean±SE. n≥4 per group. $p<0.05$ vs. *sex and age-matched WT. Statistical tests were performed using unpaired student's t tests.

Table S3: Primers for RT-PCR analysis of murine transcripts.

Name<br>**Product size (bp)**	**Forward 5-->3**	**Reverse 5-->3**
Hnf4α1/2 (95)	TGATAACCACGCTACTTGCCTT	AGCCTACTTCTGAATGTTTGGTGT
Runx2 (192)	CGGACGAGGCAAGAGTTTCA	GGATGAGGAATGCGCCCTAA
Sp7 (100)	TCTCAAGCACCAATGGACTCC	CCAGGAAATGAGTGAGGGAAGG
Bglap (146)	CCGCCTACAAACGCATCTATG	GCTGCTGTGACATCCATACTTG
Dmp1 (86)	AGTGAGGAGGACAGCCTGAA	GAGGCTCTCGTTGGACTCAC
Tnfrsf11b (287)	TGTGCTGCGCACTCCTGGTG	GGTGCGGTTGCACTCCTGCT
Tnfsf11 (223)	CTGGGACTGGGCCAGGTGGT	GTTCCTTCTGCACGGCCCCC
Rpl19 (239)	GCCCACAAGCTCTTTCCTTTCG	GTGTTTTTCCGGCAACGAGC

Table S4: Sequences of *Hnf4α* primers used to distinguish *Hnf4α* isoforms.

Isoforms	Label	Forward	Reverse	Size	Region
Total	*Hnf4α*	CTTAAGAAGTGCTTCCGGGC	CAGGCTGCTGTCCTCGTAG	99	Exon 3/4
1-3	*Hnf4α1-3*	GGAGTTTGAAAATGTGCAGGTGTTG	TGTGGTTCTTCCTCACGCTC	200	Exon 1A/2
5-6	*Hnf4α5-6*	GGAGTTTGAAAATGTGCAGGTGTTG	AACATGGTAATCGGGACAGCC	301	Exon 1A/1C
7-9	*Hnf4α7-9*	GCCTTCAGGCCCCTT	TTGAGGTTGGCACCTTCAGA	307	Exon 1D/1E
7-12	*Hnf4α10-12*	CTTTGCTGCTGTGTGTGGG	TTGAGGTTGGCACCTTCAGA	282	Exon 1E/2
1,2,4,5,7,8,10,11	*Hnf4α1/2*	TGATAACCACGCTACTTGCCTT	AGCCTACTTCTGAATGTTTGGTGT	95	Exon 10
2,5,8,11	*Hnf4α2*	GACAGATGTGTGAGTGGCCC	AGGTTACTCCCAGGTGCTCT	248	Insert 9/10
3,6,9,12	*Hnf4α3*	TTGATCCAGATGCCAAGGGG	CAGCACTACAGATCTCCCAAG	289	Exon8/ Insert8
